# A Modular Computational Approach for Assessing Active and Passive Force Contributions in Interstitial Lymphatic Fluid Uptake

**DOI:** 10.1007/s10439-026-04062-4

**Published:** 2026-03-07

**Authors:** Tharanga D. Jayathungage Don, Finbar Argus, Soroush Safaei, Peter S. Russell, Anthony RJ. Phillips, Hayley M. Reynolds

**Affiliations:** 1 Auckland Bioengineering Institute, The University of Auckland, Auckland, New Zealand; 2 School of Biological Sciences, The University of Auckland, Auckland, New Zealand; 3 Surgical and Translational Research Centre, Department of Surgery, Faculty of Medical and Health Sciences, The University of Auckland, Auckland, New Zealand

**Keywords:** Lymphatic system, Biomechanics, Initial lymphatic vessels, Active and passive forces, Valve mechanics, Computational modelling

## Abstract

**Purpose:**

The lymphatic system regulates fluid homeostasis, yet the relative roles of active vessel pumping, external mechanical forces, and lymphatic vessel/interstitium material properties on lymphatic uptake remain unclear. We sought to quantify how each factor affects lymphatic drainage.

**Method:**

We developed a bond-graph-based computational framework for modelling initial lymphatic uptake. Two networks, a simple 7-branch and a complex 19-branch architecture, were constructed to evaluate active pumping, passive external forces, and their combination. We varied vessel compliance, interstitial permeability, anchoring filament stiffness, interstitial compliance, and interstitial thickness, and performed global sensitivity analysis to identify dominant and interacting parameters. Model outputs were compared with published experimental data to assess physiological plausibility.

**Results:**

Simulated uptake rates were consistent with published ranges, supporting the model’s physiological validity. Network topology exerted strong control: the 19-branch network consistently produced lower flow than the 7-branch network. Vessel compliance increased cycle-mean flow in the 7-branch network (+11% active; + 17.8% concurrent) but reduced flow in the 19-branch network (−4.3% active; − 9.3% concurrent), indicating that structural losses in larger networks can outweigh distensibility benefits. Under passive forcing, interstitial permeability dominated drainage; very low permeability nearly eliminated flow. Anchoring filament stiffness, interstitial compliance, and thickness had negligible effects. Sensitivity analyses showed compliance governed active and concurrent force cases, whereas permeability dominated passive flow, with additional permeability/compliance interactions emerging in the 19-branch network.

**Conclusion:**

Branching topology and material properties fundamentally influence lymphatic drainage. Active pumping is particularly important in highly branched architectures, while interstitial properties mainly modulate passive flow. The framework enables quantitative exploration of regional physiological and pathological differences.

**Supplementary Information:**

The online version contains supplementary material available at https://doi.org/10.1007/s10439-026-04062-4.

## Introduction

Lymphatic research has seen increasing attention in recent years, with numerous computational models of the lymphatic system published in the last decade [1–4]. Notably, recent advances in lymphatic research have transformed perceptions of the lymphatic system. It is now recognised as more than a passive system that simply returns fluid to the circulation; it also actively contributes to fluid balance, immune regulation, and dietary fat absorption.

**Fig. 1:**
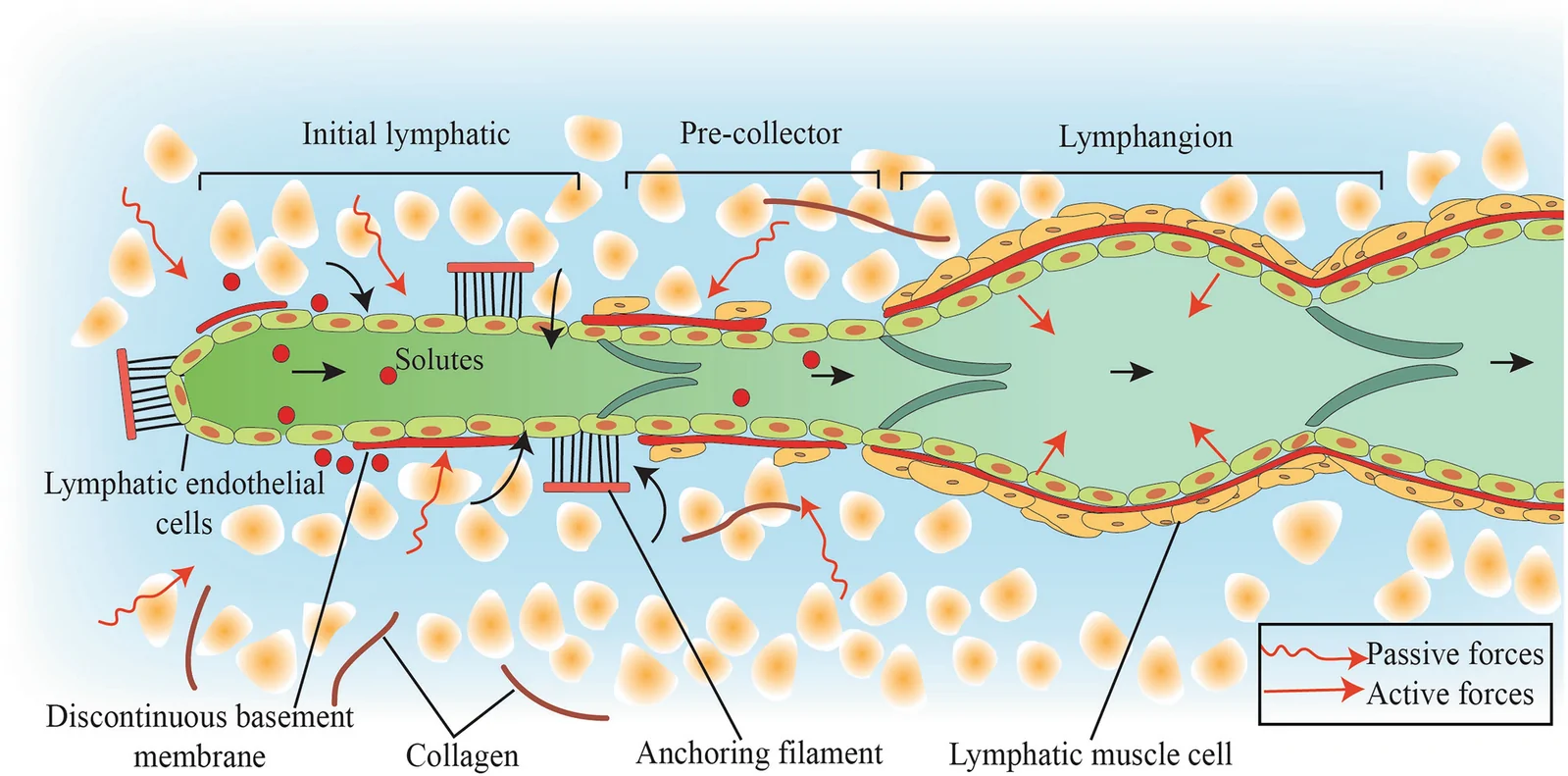
Schematic showing lymph fluid uptake (black arrows) into the initial lymphatics, pre-collecting vessels, and collecting vessels (composed of serially arranged lymphangions) that actively pump. Passive forces (wavy red arrows) indirectly drive lymphatic fluid uptake and transport, while active forces (straight red arrows) act directly to transport lymph away, causing negative pressure to facilitate initial lymphatic vessel fluid uptake

The lymphatic system comprises a hierarchy of vessels, including initial lymphatics, pre-collecting vessels, and collecting vessels (Fig. 1), which absorb lymph fluid from the interstitial space and return it to the cardiovascular system. The initial lymphatics consist of overlapping endothelial cells supported by a discontinuous basement membrane. These endothelial cells are attached to the extracellular matrix through anchoring filaments, and ‘button junctions’ between them provide gaps that permit fluid and macromolecule passage into the vessel lumen. Overlapping endothelial cells also act as one-way valves (primary valves), allowing fluid to enter the vessel and preventing its return to the interstitium [2].

Initial lymphatics connect to pre-collecting lymphatic vessels, which contain one-way valves that facilitate lymph transport to downstream collecting vessels. Collecting vessels have valves and a continuous outer layer of lymphatic muscle cells that drives active pumping, where each lymphangion (the section of vessel between two valves) acts as a 'small heart chamber', pumping lymph into the next lymphangion in series. Passive forces that drive lymphatic uptake and transport result from tissue movement (such as arterial pulsations and muscle contractions), while active forces occur via lymphangion smooth muscle contractions [5]. Despite recognising the existence of both passive and active forces in the lymphatic system, their relative importance towards how fluid enters the lymphatic lumen from the interstitial space and moves through initial lymphatic vessels is still poorly understood, highlighting a significant gap in our knowledge of lymphatic function.

Previous studies aiming to understand lymph fluid uptake from the interstitium include pioneering experiments by Guyton and Coleman [6], who revealed that interstitial pressure in most tissue beds is sub-atmospheric, which is well supported by later studies [5, 7]. In addition, Guyton et al. [6, 8] also proposed that tissue deformation from muscle contractions or arterial pulsations generates transient increases in interstitial pressure. This would facilitate fluid entering the initial lymphatics, which themselves operate at lower pressure. However, this hypothesis lacks support, as subsequent experimental studies have failed to find evidence of transient pressure spikes [9]. Jamalian et al. [9] instead demonstrated the importance of suction generated by collecting lymphatic vessels (active forces) in driving initial lymphatic uptake, addressing limitations of the interstitial pressure-driven hypothesis. Jamalian et al. [9] using combined experimental and computational approaches, showed that suction generated downstream is transmitted upstream of the secondary valve, lowering upstream pressure and drawing fluid into the initial lymphatic lumen. Several key computational modelling studies have explored lymphatic fluid uptake and transport from the interstitial space.

Reddy and Patel [10] developed the first 1D continuum model of initial lymphatics, considering 1D Poiseuille flow and wall force balance equations. They modeled lymphatic vessels as hyperelastic structures with anchoring filaments and primary valves, assuming axisymmetric flow driven under active force due to collecting vessel pumping. Their results showed that an increase in vessel length beyond a certain point did not enhance absorption. A later computational modeling study by Ikhimwin et al. [3] investigated the interaction between the interstitial space and lymphatic vessels under passive forces and showed that the mean outflow rate was sensitive to vessel stiffness. They also showed that if primary valve resistance dominates, extrinsic (external force-driven) pumping of non-rigid vessels increases the mean outflow rate. Additionally, Li et al. [11] developed an initial lymphatic drainage model embedded in a 2D poro-elastic interstitial space. They investigated the effects of interstitial pressure, downstream pressure, and tissue deformation on lymphatic uptake from the interstitial space. They concluded that the intersection angles and positions of two initial lymphatics have minimal impact on lymphatic uptake, whereas the efficiency of uptake per unit length decreases as the total length of the initial lymphatics increases.

Despite these previous studies, the relative contributions of active and passive forces to lymphatic uptake remain unclear. Therefore, in this study, we aimed to investigate the impact of these forces on lymphatic uptake from the interstitial space, focussing on anatomically accurate networks under physiologically realistic conditions.

This was implemented using bond-graph methodology, which provides a scalable and structured, physics-based representation that supports integration across spatial scales. Bond graphs allow straightforward coupling between the fluid domain and cellular mechano-chemical processes [12]. This makes them a suitable foundation for future extensions that integrate fluid dynamics with contractile lymphatic muscle cells. Bond graphs have already been used effectively to model arterial, venous, and capillary circulation [12–17], but this is the first time it has been implemented for the lymphatics. As the lymphatic system has a comparable hierarchical organisation, this framework provides a unified and extensible basis for studying lymphatic function within broader multiscale physiological models and has been used in this study to support future integration with the international IUPS Physiome Project, which is developing a comprehensive virtual human model [17].

Numerical case studies were performed using the developed lymphatic model to investigate the relative contributions of active pumping, passive force-driven flow, and their combination. For each configuration, flow rates, pressure distributions, and valve positions were analyzed under varying physiological conditions. Two lymphatic network configurations, a 7-branch and a more complex 19-branch model, were considered to evaluate the influence of anatomical complexity. A comprehensive sensitivity analysis was conducted to identify the most influential parameters affecting lymphatic transport.

## Methodology

### Lymphatic Drainage Model

Our model is based on the network framework of Ikhimwin et al. [3] and incorporates vessel compliance, bidirectional compliance coupling, and an integrated description of uptake mechanisms to advance its physiological relevance. Rather than prescribing a linear transmural pressure–diameter relationship as in Ikhimwin et al. [3], vessel deformation is governed through a mechanistic force-balance formulation that explicitly incorporates anchoring filament tension and elastic wall properties, following the principles introduced by Reddy and Patel [10]. Implementing this force balance within a spatially resolved network allows segment-level interactions between wall mechanics, interstitial loading, and intraluminal pressures to emerge dynamically.

In addition, the model incorporates compliance in both the vessel wall and the surrounding interstitium. Interstitial resistance is represented as a porous pathway, enabling bidirectional coupling between interstitial volume changes and lymphatic wall mechanics. This integrated treatment allows the model to capture how tissue hydration, stiffening, and microstructural loading influence lymphatic drainage, effects that earlier network models did not explicitly resolve.

A further capability of the present formulation is the explicit representation of multiple lymphatic uptake mechanisms. The framework allows passive pressure-driven uptake, active pumping, and their combined action to be simulated under identical network geometry and parameters. This advances beyond Ikhimwin’s model, which considered only passive force-driven uptake. This enables direct, mechanism-specific comparison of drainage behavior across physiological and pathophysiological boundary conditions. Together, these extensions enable physiologically relevant simulations beyond the scope of earlier formulations.

**Fig. 2:**
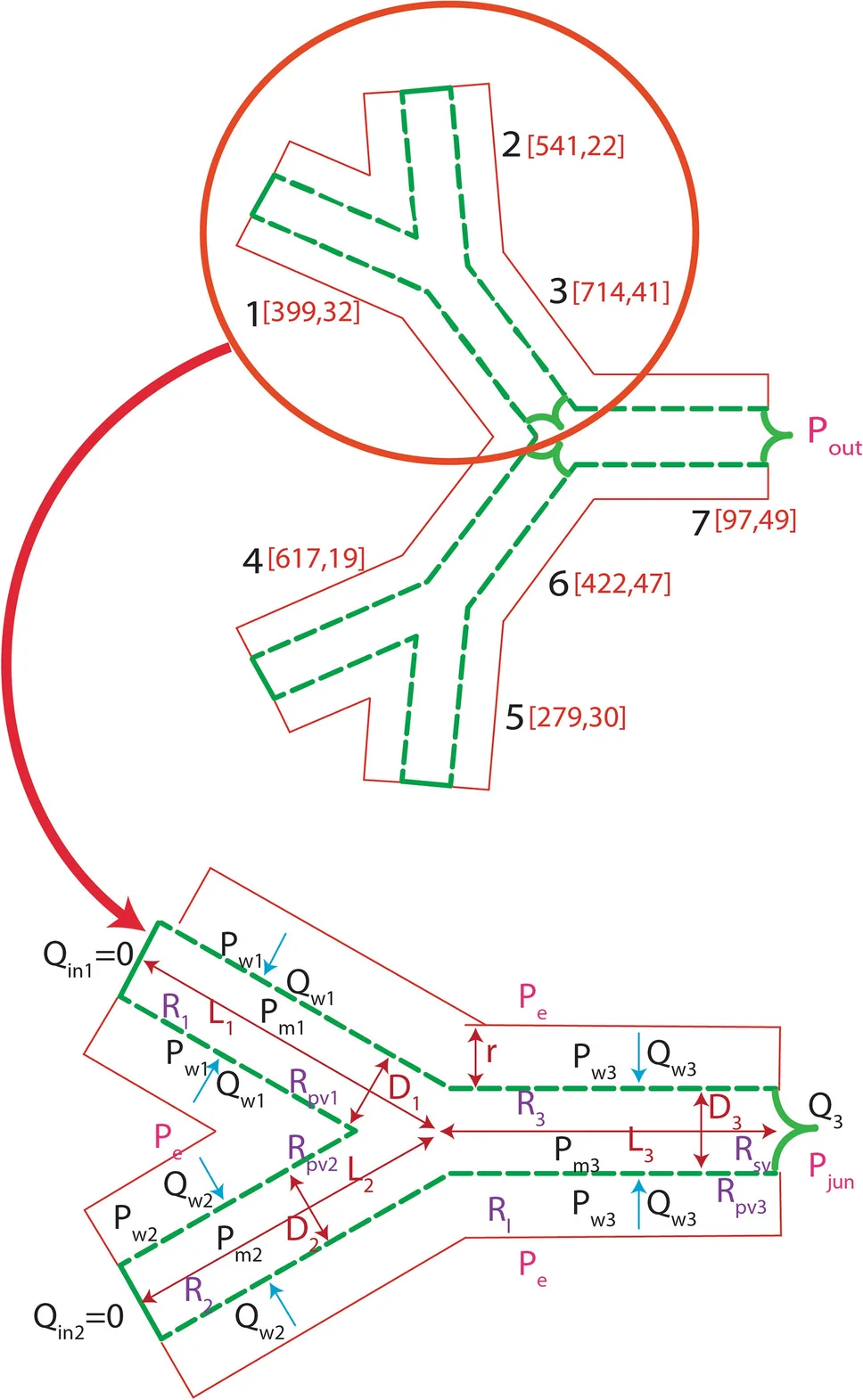
Schematic representation of the 7-branch network model. Initial lymphatic vessels are shown as discontinuous green lines, indicating primary valve junctions between endothelial cells. Each vessel is assumed to reside within a porous interstitial cylinder, represented by red lines. Flow rate (Q), pressure (P), and resistance (R) are annotated, with subscripts denoting external pressure at the interstitium (e), wall (w), mid-vessel (m), primary valve (pv), interstitium (I), and secondary valve (sv). The length (L) and diameter (D) of the vessel segments vary between branches, shown in square brackets. The diameter and length values are based on rat mesenteric lymphatic vessel data [19]

Bond graph modules were developed for the initial and pre-collecting lymphatic vessels with primary valves, secondary valves, and the surrounding interstitium. Figure 2 schematically illustrates a 7-branch network model that was generated. Original imaging data were obtained from Murfee et al. [18], with lengths and diameters later estimated by Ikhimwin [19]. The initial lymphatic vessels are shown as discontinuous green lines, representingdiscontinuous junctions between endothelial cells (the ‘primary valves’). We assumed that each vessel lies within a porous interstitial cylinder, as above. Flow rate (Q), pressure (P), and resistance (R) are denoted, with subscripts indicating external pressure at the interstitium (e), wall (w), mid-vessel (m), primary valve (pv), interstitium (I), and secondary valve (sv). The length (L) and diameter (D) of vessel segments differ across branches.

Due to the low Reynolds and Womersley numbers in the initial and pre-collecting lymphatics, we neglect inertial effects. The flow is modelled assuming a fully developed, parabolic profile, giving Poiseuille's equation [3, 20–25]. Lymph fluid was assumed to be an incompressible Newtonian fluid flowing through compliant vessels. Each anatomical vessel was represented by two lumped compartments of equal length, with a mid-point node at which interstitial fluid enters the lumen via primary valves. Thus, a physical segment of length L was modelled as two computational half-segments, each of length L/2. The axial flow in each half-segment is described by a Poiseuille relation:

1 $$Q=\frac{\pi {D}^{4}}{\frac{64\mu L}{\Delta P}},$$

where ΔP is the pressure difference in the vessel, and µ is the viscosity of the lymph fluid. For initial blind-ended branches, the proximal half-segment has zero axial inflow (no upstream vessel), and all lymph entering that branch arises from interstitial drainage through the wall at the mid-point. In this quasi-steady approximation, we neglect fluid inertial effects but retain time-dependent deformation of the vessel wall and interstitium; at each time point, the geometry is updated by the compliance model, and the corresponding flow rate is computed. The flow in the interstitial space, which surrounds the initial lymphatic vessels, was modeled using Darcy’s law:

2 $$q(r)=\frac{K}{\mu }\frac{dp}{dr},$$

where $\frac{dp}{dr}$ is the pressure gradient in the radial direction, q is the flow rate per unit area in the radial direction, and K is the permeability of the interstitial space. The total flow draining into an initial lymphatic vessel, which has length L, is calculated by integrating with respect to p and r to give:

3 $$R=\frac{\mu (ln(({2r}_{I}+D)/D))}{2\pi LK}$$

4 $${Q}_{I}={Q}_{w}=\frac{({P}_{e}-{P}_{w})}{R},$$

where Q_I_ is the flow rate through the interstitium that drains into the lymphatic vessels, which is equal to Q_w_. Additionally, the interstitial resistance value, which can be estimated based on Equation 3, is obtained from the ratio Q_I_/ (${P}_{e}-{P}_{w}$). ${r}_{I}$ is the interstitial thickness and D is the vessel diameter.

Initial lymphatic vessels were considered deformable with external wall anchors and external pressure acting on the vessel wall. By assuming the initial lymphatic vessels are thin, cylindrical vessels, the force balance equation can be written as follows:

5 $${P}_{m}={P}_{w}+\frac{2h}{D}\sigma -\frac{{F}_{A}}{\pi DL},$$

where P_m_ represents the mid-vessel pressure (halfway point along the vessel length), P_w_ denotes the external pressure on the vessel wall, F_A_ stands for the force applied to the vessel wall by the anchoring filaments, σ is the hoop stress in the vessel wall, and h represents the thickness of the vessel wall. Notably, P_w_ and F_A_ exert forces in opposite directions. The mechanical hoop stress term is replaced with the flow-compliance relationship, and it is depicted in Equation A.16. F_A_ can be calculated as [10]:

6 $${F}_{A}=N{K}_{AF}({L}_{f}-{L}_{f0}),$$

where N is the number of filaments per unit length, K_AF_ is a constant related to the elastic properties of anchoring filaments (similar to a spring constant), L_f_ is the inter-fibre distance, and L_f0_ is the initial inter-fibre distance length which is 2.5 times the initial diameter (D_0_) [10]. The filament length L_f_ depends on the inter-fibre distance and the vessel wall’s instantaneous diameter (D), which is influenced by the interstitial fluid volume. Based on the Reddy and Patel [10] model, F_A_ can be calculated using:

7 $${F}_{A}=N{K}_{AF}\left(\frac{{D}_{0}-D}{2}\right)\left(0.5\times \frac{{L}_{f0}}{\left({r}_{I}-{D}_{0}\right)}+1\right)$$

Derivation of Equation 7 is discussed in Section A.1.2.1 in the supplementary materials.

#### Primary valves

Primary valves were modeled using the following equation:

8 $${R}_{pv}=\frac{H{R}_{pv0}}{\frac{2\pi LD}{d}\bullet \left[cosh\left[a\bullet \Delta {p}_{pv}\right]-1\right]\bullet {e}^{{-b\bullet \Delta p}_{pv}}}$$

R_pv_ represents the primary valve resistance, and R_pv0_ is a conversion constant. The transmural pressure, ΔP_pv_ = P_m_ - P_w_, is the pressure difference between the mid-vessel pressure (P_m_) and the pressure just outside the wall (P_w_). For ideal primary valves, R_pv_ becomes infinite when P_m_ > P_w_. This equation was previously derived by Ikhimwin et al. [3] who modeled the primary valve resistance in initial lymphatic vessels by fitting it to pressure drop versus flow rate curves from an earlier study by Galie and Spilker [26]. H denotes Horsfield order, which is the number that indicates the generation levels of any network segment. Each Horsfield order (H) then modifies the primary valve resistance ([3]; see Equation 8), as the primary valve resistance is higher in pre-collectors.

#### Secondary Valves

A simplified model of secondary valve function was used by incorporating a step function to simulate the valve's behavior. The valve state (open or closed) is determined by comparing upstream and downstream pressures, as defined in Equation 9.

9 $${R}_{sv}=\left\{\begin{array}{c}0.025 dyn/{s}^{-1}{cm}^{5} if \Delta {P}_{valve} \ge 0 \\ {10}^{30} dyn/{s}^{-1}{cm}^{5} if \Delta {P}_{valve} < 0\end{array}\right.$$

#### Lymphatic Bond Graph Model

All model equations are converted into a bond graph formulation, providing a modularised and scalable framework capable of simulating lymph flow dynamics in anatomical networks of lymphatic vessels. Further information about bond graph methodology, this conversion process, and bond graph formulations is explained in the supplementary materials. Note that we have used conventional physiological symbols ***P*** (pressure), ***Q*** (flow rate), and ***R*** (resistance) here to improve readability; however, standard bond-graph notation (effort = *u*, flow = *v*, generalised displacement/charge = *q*) is used in the Supplementary materials.

**Fig. 3:**
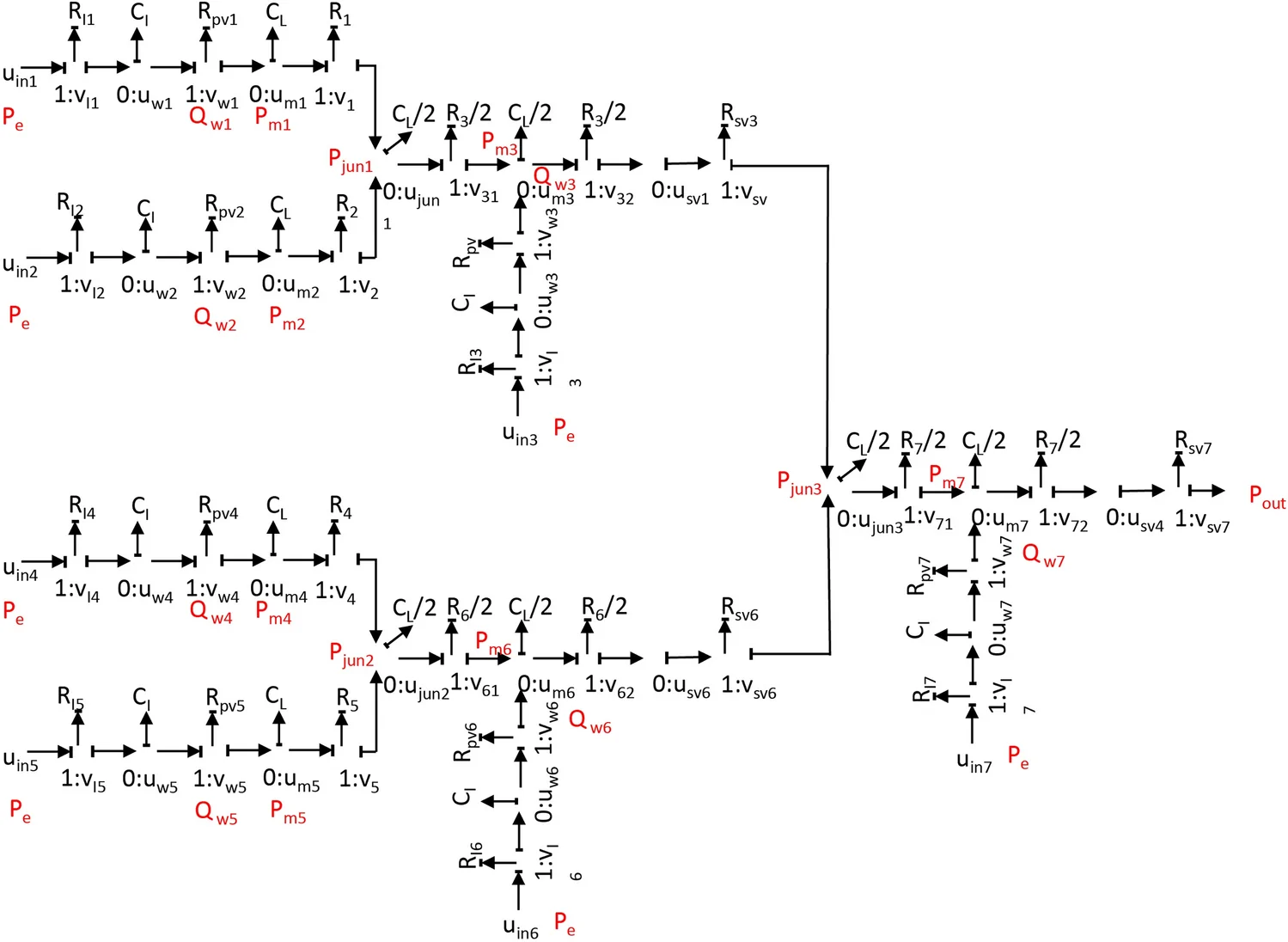
Bond graph representation of the 7-branch lymphatic model. In this representation, '*u*' signifies pressure (*P*) and '*v*' signifies lymph fluid flow (*Q*) in their respective branches. The vessels are labelled 1 to 7, with 'm' indicating the mid-point of each vessel. Conventional flow (*Q*) and pressure (*P*) variables in the original 7-branch network shown in Fig. 2 are highlighted in red. Interstitial, vessel, primary valve, and secondary valve resistances are denoted as *R*_*I*_*-1-7, R*_*pv-1-7*_*, R*_*-1-7*_*, R*_*pv1-7,*_* and R*_*sv-3,6,7*,_ respectively

Flow dynamics were first modelled in a simple 7-branch network with three generations considered (Fig. 2) using topological data extracted from rat mesenteric lymphatic vessels reported in Murfee et al.[18] and Ikhimwin [19]. Each lymphatic vessel segment and the flow through the wall were represented using bond graph elements and junctions (Fig. 3), which track mass flows in accordance with the mass conservation principle. The initial lymphatic vessels were assumed to have blind ends, preventing lymph fluid from entering through the vessel ends; thus, lymph fluid can only enter through the porous walls of these four vessel branches (see branches 1, 2, 4, and 5 in Fig. 2).

Three bond graph modules were developed to generate a system of ordinary differential equations (ODEs), as detailed in the supplementary materials. These included a module for blind-ended initial lymphatic vessels, a second for mid-lymphatic vessels (representing either pre-collectors with valves or valve-less initial lymphatics), and a third for outer collecting lymphatic vessels. Modules are connected according to the branching network data, enabling a scalable framework using Circulatory Autogen [27]. ODEs were encoded in the open-source, extensible markup language (XML)-based CellML modeling environment [28]. All simulations were conducted using OpenCOR, an open-source, cross-platform modeling environment that supports CellML models [29].

### Case Studies

**Fig. 4:**
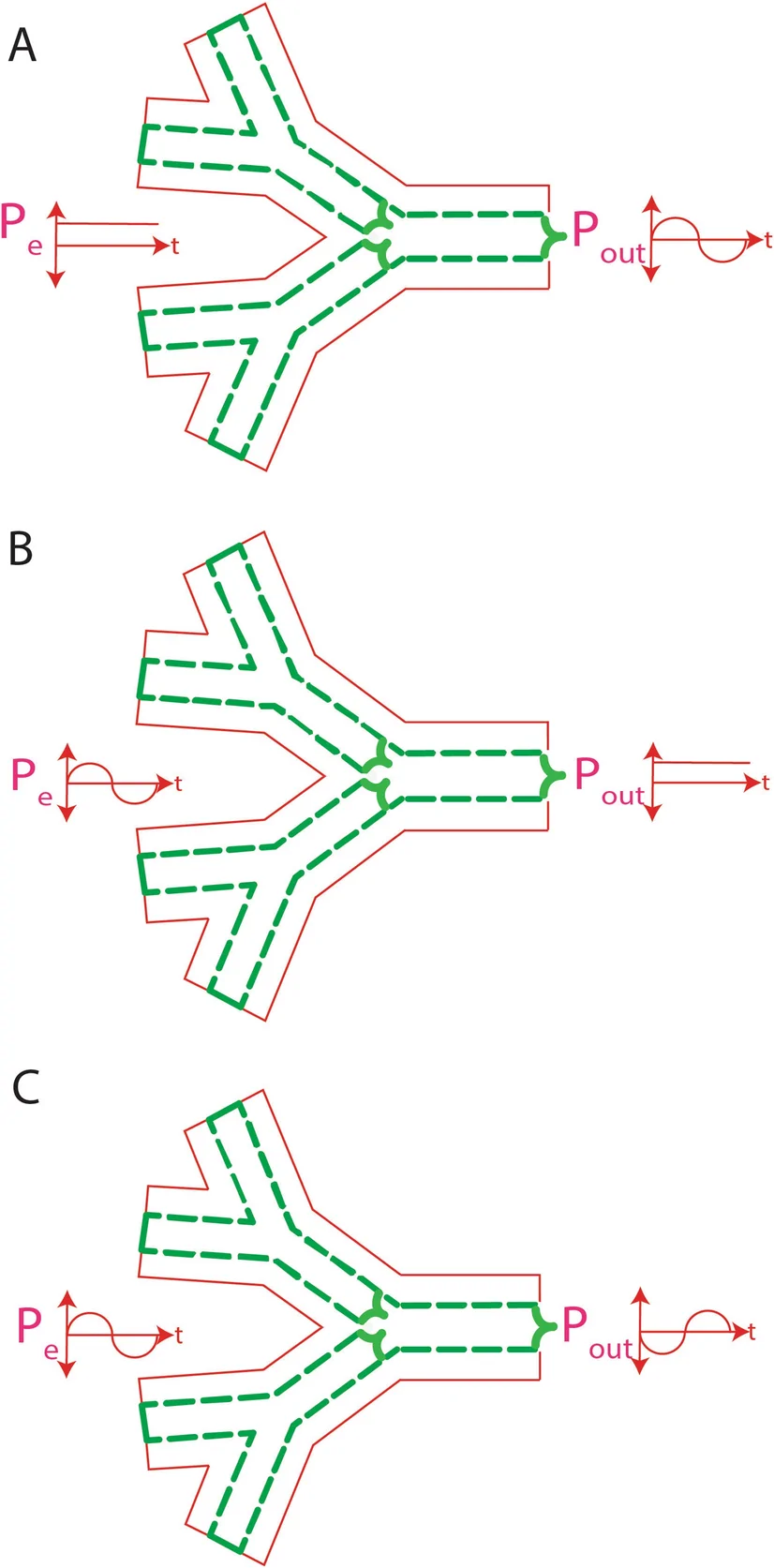
Schematic representation of the three case studies, covering A: active force-driven lymphatic drainage, considering constant pressure in the interstitium and time-varying pressure conditions at the vessel outlet; B: passive force-driven lymphatic drainage, considering constant pressure at the vessel outlet and time-varying pressure conditions in the interstitium; and C: concurrent active and passive force-driven lymphatic drainage, considering time-varying pressure conditions at both the interstitium and vessel outlet

Three different case studies were considered, as demonstrated in Fig. 4, aiming to understand the individual and concurrent effects of passive and active forces on lymphatic uptake. The first case study considered active force-driven lymphatic uptake (Fig. 4A), the second considered passive force-driven lymphatic uptake (Fig. 4B), and the third considered combined active and passive force-driven lymphatic uptake (Fig. 4C). Additionally, a model parameter sensitivity analysis was performed for each case study, and a complex extended network simulation was conducted to demonstrate the scalability of the modularised bond graph approach.

#### Active Force-Driven Lymphatic Uptake

Active force-driven lymphatic uptake is primarily driven by the contractions of collecting lymphatic vessels [9]. Model boundary conditions included time-varying pressure at the outlet of branch 7 (see Fig. 2) to mimic lymphatic muscle cell contraction in the downstream collecting lymphatic vessel. The boundary condition at the outlet, P_out_, was:

10 $${P}_{out}=\frac{A}{2}\left(3-cos2\pi {f}_{A}t\right),$$

where A is the amplitude of the pressure wave, t is the time, and f_A_ is the frequency of the active force. A constant *P*_*e*_ value was imposed at the interstitial side. *P*_*out*_ yields a minimum, maximum, and mean pressure of A_P_, 2A_P_, and 1.5 A_P_, respectively. Setting A_p_ = 98 Pa (approximately 1 cmH₂O), ***P***_***out***_ therefore oscillates between about 98 Pa and 196 Pa, with a mean of 147 Pa.

#### Passive Force-Driven Lymphatic Uptake

Passive force-driven lymphatic uptake arises from interstitial pressure fluctuations generated by tissue deformation. To represent physiologically driven interstitial pulsations, we applied a spatially uniform sinusoidal pressure across the lymphatic network, assuming that the surrounding interstitium behaves as a large-scale moving boundary relative to vessel dimensions. The outlet pressure at branch 7 was fixed at 147 Pa, consistent with the active force-driven case. The sinusoidal form is a simplified representation of the dynamic pressure variations produced by arterial pulsations, skeletal muscle contractions, intestinal peristalsis, lung ventilation, cardiac motion, and transient external loading. The passive pressure waveform was defined as

11 $${P}_{e}=\frac{A}{2}\left(3-cos2\pi {f}_{P}t\right),$$

where f_P_ is the frequency of the passive force, and the outlet pressure, P_out_, was considered constant.

#### Concurrent Active and Passive Force-driven Lymphatic Uptake

To simulate the combined effects of active and passive forces (Equations 10 and 11), the phase differences (t_1_) between them were considered. Both time-varying pressure at the interstitium (Equation 11) and outlet (Equation 12) with phase difference (t_1_) were considered. Therefore, P_out_ was imposed by:

12 $${P}_{out}=\frac{A}{2}\left(3-cos2\pi {f}_{A}\left(t-{t}_{1}\right)\right)$$

#### Complex Extended Network Simulation

**Fig. 5:**
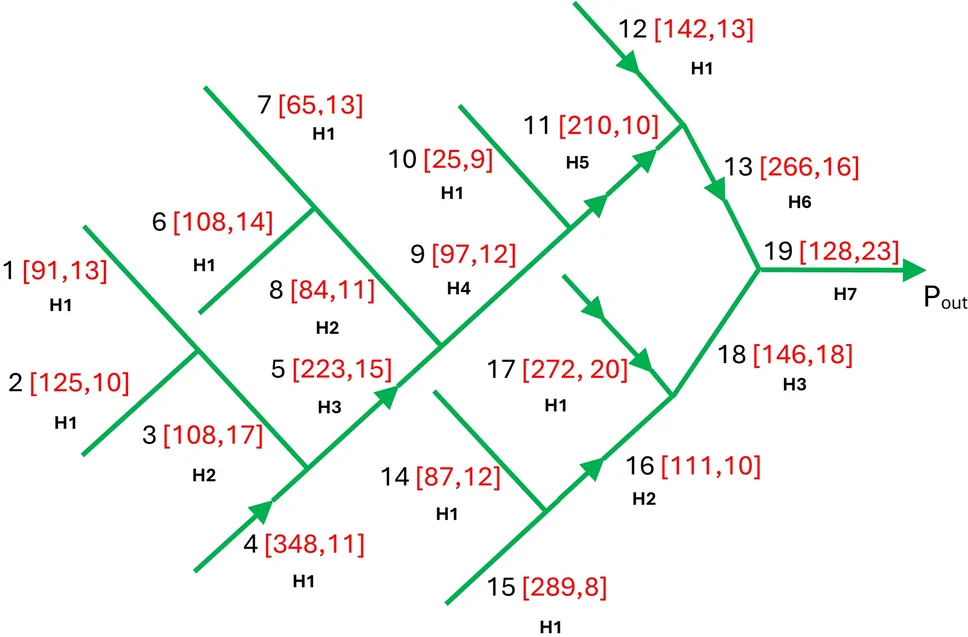
Schematic diagram of the 19-segment network, with branch lengths and diameters [L, D] depicted in brackets obtained from the initial lymphatic network of a rat mesentery [19]. H 1-7 shows the Horsfield generation number of each segment. Green triangles represent the secondary valves

To model a more complex extended lymphatic network, data from a study by Ikhimwin [19], which reported length and diameter measurements for a 19-branch rat mesentery vessel network, were used. Figure 5 depicts the complex initial-pre-collecting network consisting of 19 branches, some of which include secondary valves. Also, in Fig. 5, H1–7 indicates the Horsfield generation number assigned to each segment. Equation 8 represents the primary valve resistance, which incorporates the Horsfield generation number. To ensure a fair comparison of lymphatic drainage, we have used matching H values (extracted by going backward from the outlet branch of Fig. 5) in the 7-branch network. The network topology was converted into a bond graph model by connecting multiple modules using Circulatory Autogen software [27] (for more details, see A.2-4). This tool was developed to integrate CellML modules into system models that reflect anatomical and functional data from clinical or experimental sources. The number of secondary valves in each branch determined differences in the defined bond graph modules. For instance, the lymphatic mid-vessel model defined here has two valves (See Section A.2.2), whereas a mid-vessel branch that only has one secondary valve requires a bond graph module with one fewer R_sv_ term.

Compared with the simplified 7-branch, this network includes smaller average vessel diameters and shorter, more heterogeneous segments, providing a more anatomically detailed test case. Including this larger network allows us to examine how the modelling framework behaves in a geometry that better reflects the complexity of the initial lymphatic network observed in imaging data.

#### Parameter Values

Table 1 lists the parameter values used and their sources, primarily derived from rodent mesenteric lymphatic vessels. The frequencies of active and passive contractions (f_A_ and f_P_) were both set at 0.5 Hz and are from Ikhimwin et al. [3] and Reddy and Patel [10].

**Table 1:** Model parameters

Parameter	Description	Value	Unit	Reference
E	Young’s modulus	42	kPa	[10]
h	Thickness of the vessel wall	0.001	mm	[10]
µ	Fluid Viscosity	0.001	kgm^-1^ s^-1^	[3]
f_A_	Frequency of active contractions	0.5	Hz	[30]
f_P_	Frequency of passive pressure	0.5	Hz	[3]
P_out_	Outlet pressure	147 or Equations (10)/(12)	Pa	[31]
P_e_	Interstitial pressure	147 or Equation (11)	Pa	[3]
r_I_	Radius (thickness) of the interstitium	0.0003	m	[3]
A	Amplitude	98.07	Pa	[3]
t_1_	Phase difference	90	degrees	
N	Number of anchoring filaments per m	10^6^	m^-1^	[10]
L_f0_	Initial inter-fibre distance	2.5 × resting diameter	m	[10]
C_L_	Vessel wall compliance	1.88 × 10^-16^	m^3^Pa^-1^	[32]
C_I_	Interstitial space compliance	1.52 × 10^-14^	m^3^Pa^-1^	[5, 33]
K	Interstitial space permeability	9.60 × 10^-12^	m^2^	[34]
K_AF_	The spring constant of anchoring filaments	9.43 × 10^-6^	N/m	[10]
R_pv0_	Unit conversion constant	10^3^	kgm^-3^ s^-1^	[3]
a	Fit constant for primary valve model	7.88 × 10^-7^	Pa^-1^	[3]
b	Fit constant for primary valve model	7.11 × 10^-4^	Pa^-1^	[3]
d	Characteristic endothelial cell dimension	2	µm	[3]

#### Parameter Sensitivity Study

A parameter sensitivity study was conducted to assess the influence of key parameters (see Table 2) on the total lymphatic drainage, defined by the cycle-averaged outflow rate. This analysis was conducted for each of the three cases to assess the model's robustness and critical dependencies under varying physiological conditions.

**Table 2:** Baseline Values and Sensitivity Study Ranges for Key Model Parameters

Parameter	Symbol/(unit)	Baseline value	Minimum value	Maximum value	Reference
Vessel wall compliance	C_L_ /(m^3^Pa^-1^)	1.88 × 10^-16^	1.00 × 10^-16^	2.76 × 10^-16^	[32]
Interstitial space compliance	C_I_ /(m^3^Pa^-1^)	1.52 × 10^-14^	9.10 × 10^--16^	5.56 × 10^-14^	[5, 33]
Interstitial space permeability	K /(m^2^)	9.60 × 10^-12^	1.00 × 10^-15^	1.00 × 10^-11^	[34]
Radius (thickness) of the interstitium	r_I_ /(m)	3.00 × 10^-4^	5.00 × 10^-5^	5.00 × 10^-4^	[36]
Spring constant of anchoring filaments	K_AF_ /(N/m)	9.43 × 10^-6^	2.35 × 10^-6^	2.12 × 10^-5^	[10, 36]

The baseline vessel compliance parameters were derived from experimental measurements and converted from specific compliances (fractional volume change per pressure) to absolute compliances using the model's reference volumes to maintain physiological scaling. Specifically, interstitial quantities were scaled by the average interstitial volume (V_I_ = 3.76 × 10^-11^ m^3^), and vessel quantities by the average vessel volume (V_L_ = 3.93 × 10^-13^ m^3^).

Vessel Wall Compliance (C_L_, m^3^/Pa): The baseline specific compliance (7.0 × 10^^-4^ Pa^-1^) was adopted from the work of Negrini and Moriondo [32], representing the average of two distinct vessel populations identified in rat diaphragmatic initial lymphatics: low-compliance/stiff vessels (2.55 × 10^-4^ Pa^-1^, embedded in muscle) and high-compliance/compliant vessels (1.14 × 10^-3^ Pa^-1^, in soft connective tissue). By multiplying these specific compliances by the average vessel volume (V_L_), the minimum, baseline, and maximum absolute compliance values were estimated for the sensitivity analysis (Table 2).

Interstitial Compliance (C_I_, m^3^/Pa): The baseline interstitial compliance was set to 1.52 × 10^-14^ m^3^/Pa, converted from values reported by Wiig and Reed [33] for rat-skin interstitial compliance (~5.4 ml per 100 g wet tissue per mmHg) and scaled by the interstitial volume (V_I_). To accurately model the known non-linear pressure–volume behavior of the interstitium, a range spanning 0.06 to 3.7 times the nominal value was utilised [5, 33]. The lower bound of this range represents dehydrated tissue with low interstitial fluid volume, while the upper bound corresponds to edematous conditions where substantial volume accumulation occurs with only modest increases in interstitial fluid pressure.

Interstitial Permeability (K, m^2^): The baseline hydraulic conductivity was taken from in-vitro measurements of mouse tail skin reported by Swartz et al. [34] (9.6 × 10^-12^ m^2^). The sensitivity study range, 1 × 10^-15^ to 1 × 10^-11^ m^2^, was applied to the permeability values reported by Salavati et al. [35], which accounts for the increase in permeability observed during conditions such as edema.

Interstitial Radius/Thickness (r_I_, m): A baseline effective drainage radius of 300 µm was set, with bounds of 50 to 500 µm. This range reflects the reported variation in lymphatic density and spacing across different tissues, such as the diaphragm and skin [18, 36].

Anchoring Filament Stiffness (K_AF_, N/m): In the absence of direct experimental measurements, the nominal stiffness was estimated to be 9.43 × 10^-6^ N/m per filament, based on theoretical filament mechanics (material modulus, length, and nominal diameter). The bounds for the sensitivity analysis were derived by assuming that stiffness is proportional to the filament's cross-sectional area (K_AF_ proportional to d^2^), corresponding to a diameter range of 20 to 60 nm [10, 37] gives an anchoring filament stiffness range of approximately 0.25 to 2.25 times the baseline value.

## Sobol Variance-Based Global Sensitivity Analysis

A Sobol variance-based global sensitivity analysis was performed on the cycle-mean outflow rate, measured at the outlet branch (branch 7 in the simple network and branch 19 in the complex network), for the five parameters in Table 2. This was conducted using the Circulatory Autogen sensitivity module [27] which decomposes the variance of the model output into contributions from individual parameters and their interactions, providing a robust measure of parameter influence. Quasi-random Sobol sequences were used to generate parameter samples, and for each sample, the lymphatic network model was solved to compute the resulting cycle-mean outflow at the outlet branch (branch 7 in the simple network and branch 19 in the complex network).

Both first-order Sobol indices and total-order indices were estimated to distinguish parameters with strong direct effects from those involved in higher-order interactions. The analysis was repeated independently for the passive, active, and combined pumping conditions to assess whether parameter influence differed across physiologically distinct loading scenarios. Additional information is provided in the Supplementary Material.

## Results

Total lymphatic drainage, which is the flow rate at the outlet branch of the simple 7-branch network and complex 19-branch network models for each of the three case studies, was computed and compared.

### Active Force-Driven Lymphatic Uptake

**Fig. 6:**
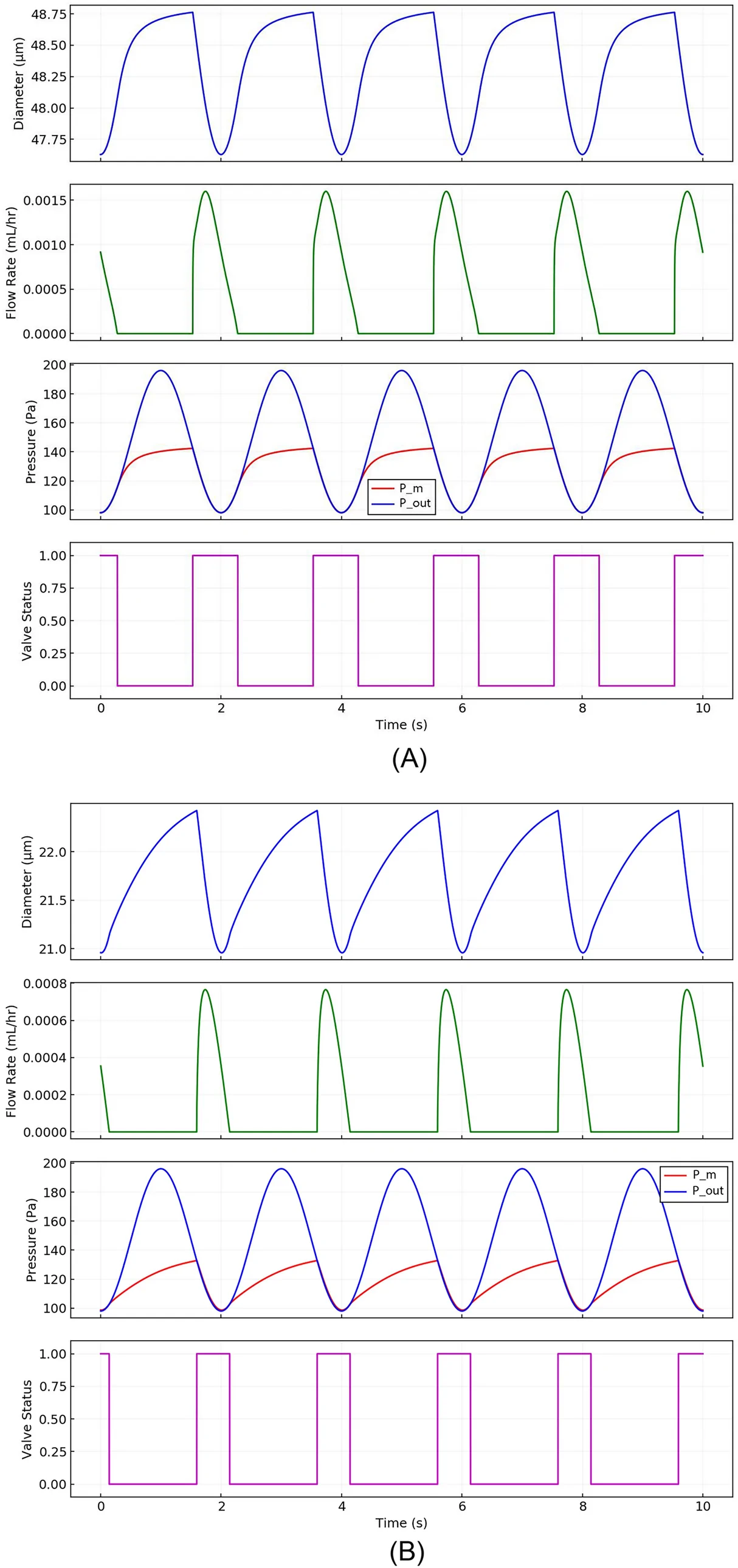
Active force-driven lymphatic uptake results showing the outlet vessel diameters, lymph fluid flow rates, mid pressures, and valve status (open-1/closed-0) within **A** outlet branch 7 in the simple 7-branch network and **B** outlet branch 19 in the complex 19-branch network

Figure 6 plots the vessel diameter, lymph fluid flow rate, pressure, and valve status in the outlet branch caused by active forces in the 7-branch (Fig. 6A) and 19-branch (Fig. 6B) networks. The valve opens (1) when the mid pressure surpasses the pressure at the outlet and vice versa, and the presence of the secondary valve permits one-way flow in the outlet branch. Consequently, the resulting outlet flow rates exhibit pulsatile characteristics with cycle-mean and maximum flow rates of 3.69 × 10^-4^ ml/hr and 1.6 × 10^-3^ ml/hr for the 7-branch network and 1.37 × 10^-4^ ml/hr and 7.8 × 10^-4^ ml/hr for the 19-branch network, respectively. Mid-vessel pressure closely follows the sinusoidal outlet pressure profile when the valve is opened. The valve stays open longer in the outlet branch of the 7-branch network compared to the 19-branch network.

#### Parameter Sensitivity Analysis

**Fig. 7:**
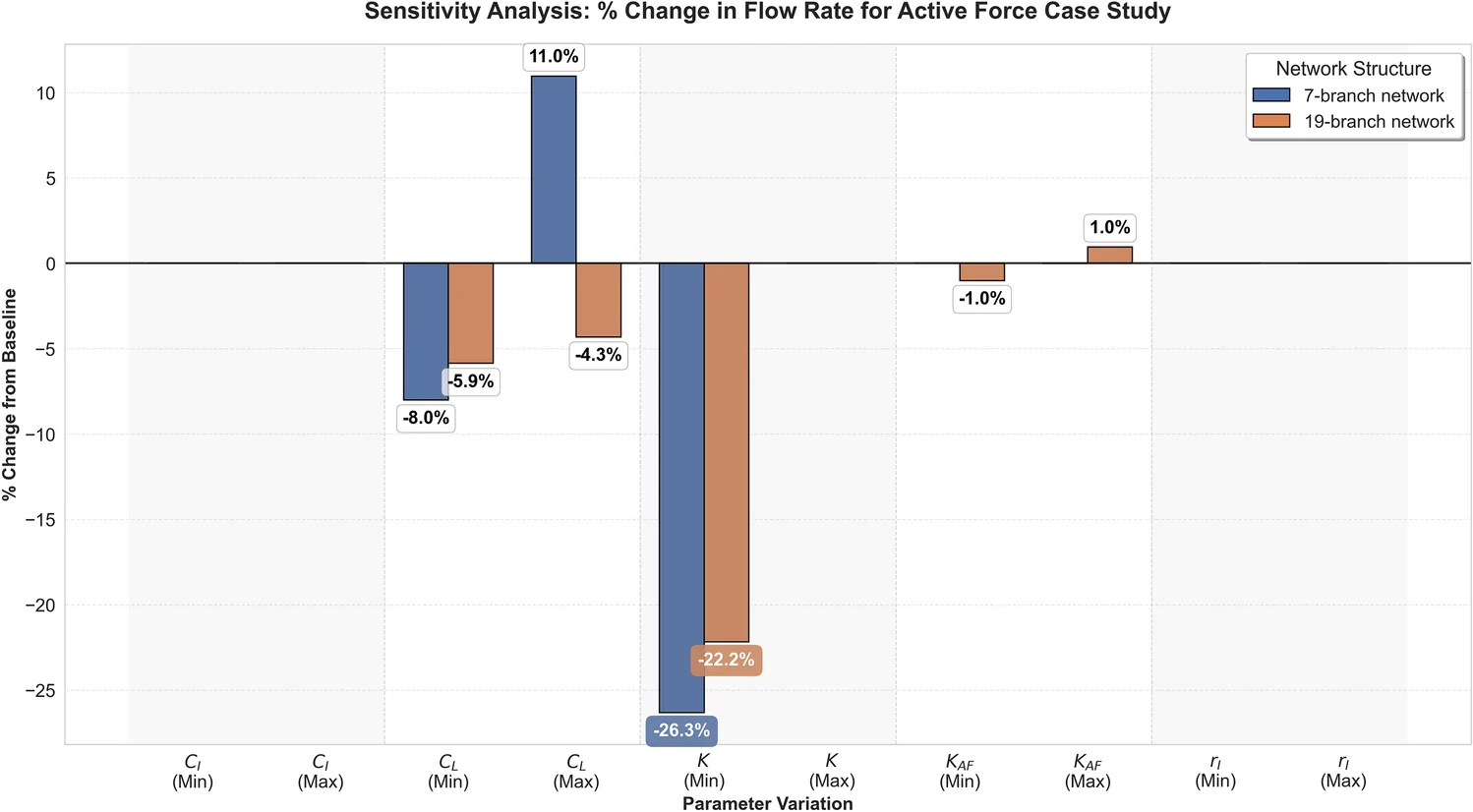
Parameter sensitivity analysis results for the active force-driven lymphatic uptake case study, showing the % change in flow rate relative to baseline

Figure 7 shows the sensitivity analysis results from the active force case study, showing the percentage change in total lymphatic flow rate relative to the baseline. Flow values are normalised to the baseline case to allow direct comparison across parameter variations. Interstitial permeability (K) was the most influential parameter, causing a flow reduction of 26.3% (7-branch) and 22.2% (19-branch) at its minimum value (Table 2). Vessel wall compliance (C_L_) was the second-most-influential parameter, with a flow increase of 11.0% in the 7-branch network at its maximum value. The remaining parameters C_I_, K_AF_, and r_I_, had a negligible impact, with flow changes all below 1%.

### Passive Force-Driven Lymphatic Drainage

**Fig. 8:**
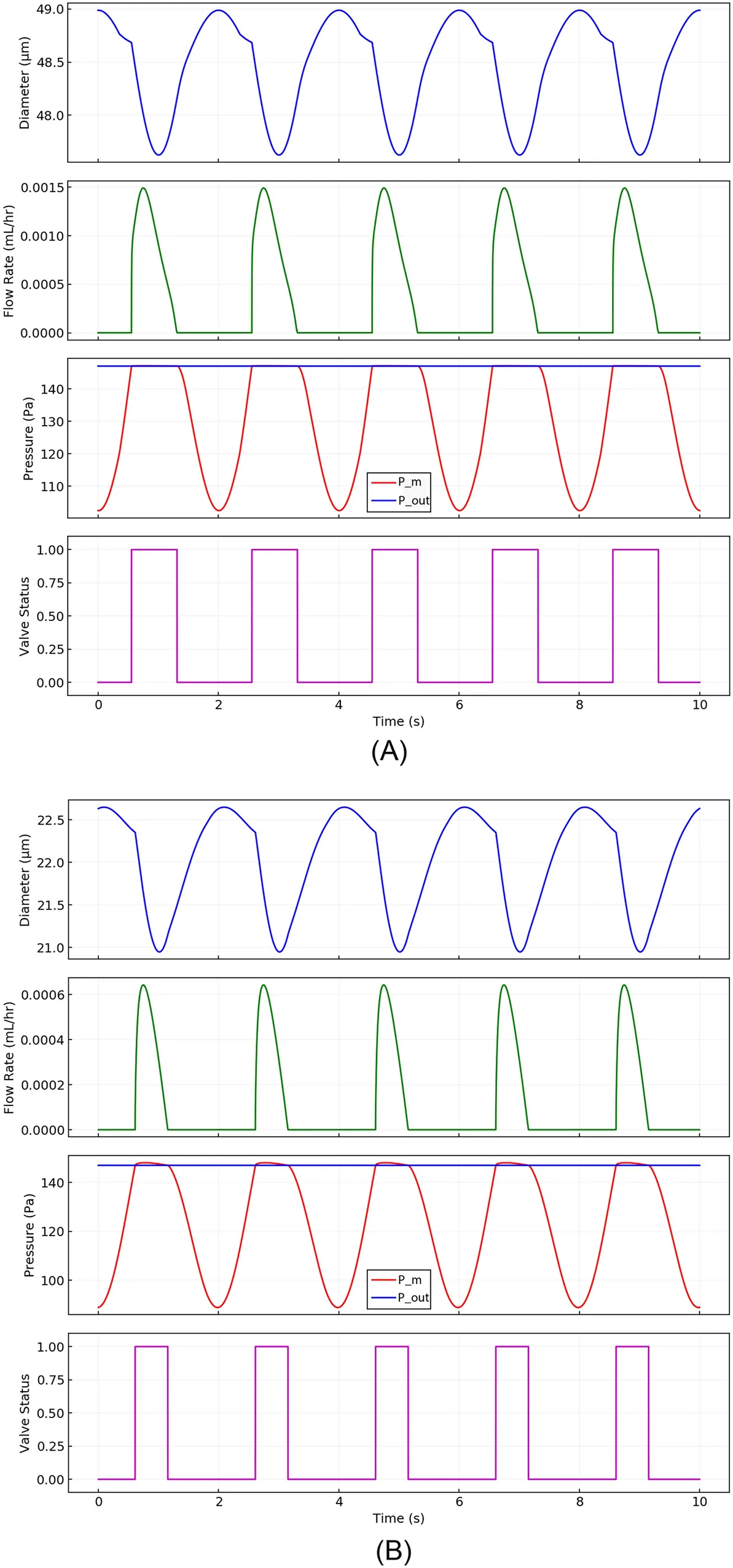
Passive force-driven lymphatic uptake results for P_out_ = 147 Pa. The figures show outlet vessel diameters, lymph fluid flow rates, mid pressures, and valve status (open-1/closed-0) within **A** outlet branch 7 in the simple 7-branch network and **B** outlet branch 19 in the complex 19-branch network

Figure 8 plots the vessel diameter, lymph fluid flow rate, pressure values, and valve status in the outlet branch due to passive forces in the interstitial space, in the 7-branch (Fig. 8A) and 19-branch (Fig. 8B) networks. In the 7-branch network (P_out_ = 147 Pa), the cycle-mean and maximum flow rates were 3.56 × 10^-4^ ml/hr and 1.5 × 10^-3^ ml/hr, respectively. There was a 6% reduction in the cycle-mean flow rate compared to the active force-driven lymphatic drainage. The mid-vessel pressure (P_m7_) in Fig. 8A and B show sinusoidal pressure variation with a plateau at 147 Pa.

Figure 8B shows the passive force-driven lymphatic uptake results for the 19-branch network. The cycle-mean and maximum flow rates were 1.13 × 10^-4^ ml/hr and 6.5 × 10^-4^ ml/hr, respectively. Similar to the previous case study, the valve stays open longer in the 7-branch case study.

#### Parameter Sensitivity Analysis

**Fig. 9:**
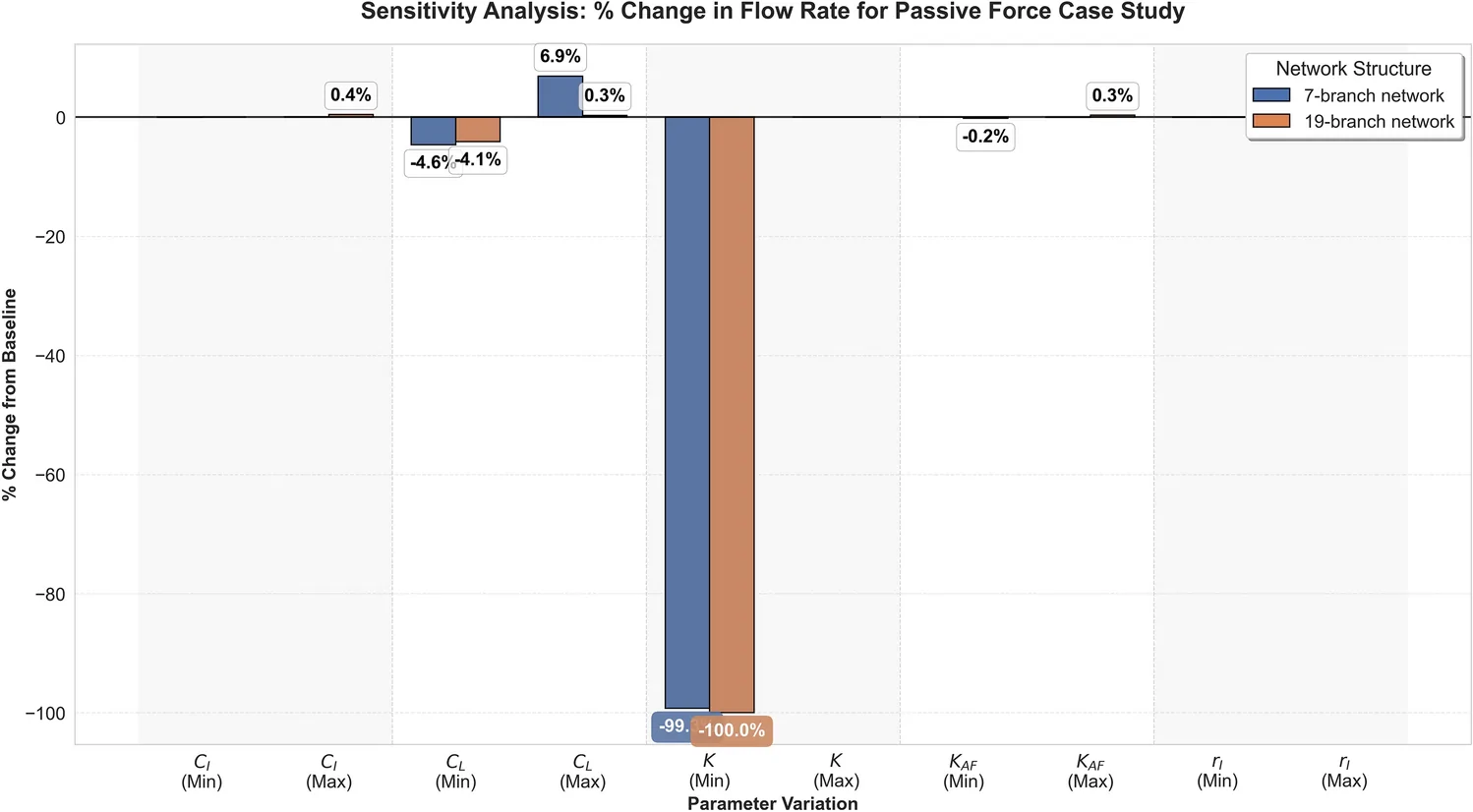
Parameter sensitivity analysis results for the passive force-driven lymphatic uptake, showing the % change in flow rate relative to baseline

Figure 9 shows the parameter sensitivity results for the passive force case study, plotting total lymphatic drainage compared to the baseline. The outflow rate was most sensitive to the interstitial space permeability (K), where the minimum K value resulted in a 99.3% reduction in flow for the 7-branch network and a 100% reduction for the 19-branch network. The second most influential parameter was C_L,_ which caused an increase in flow of 6.90% at its maximum value in the 7-branch network and a decrease in flow of 4.10% at its minimum value in the 19-branch network. The remaining parameters, C_I_, K_AF_, and r_I_, had a negligible impact, with flow changes consistently below 1%.

### Concurrent Active and Passive Force-Driven Lymphatic Uptake

**Fig. 10:**
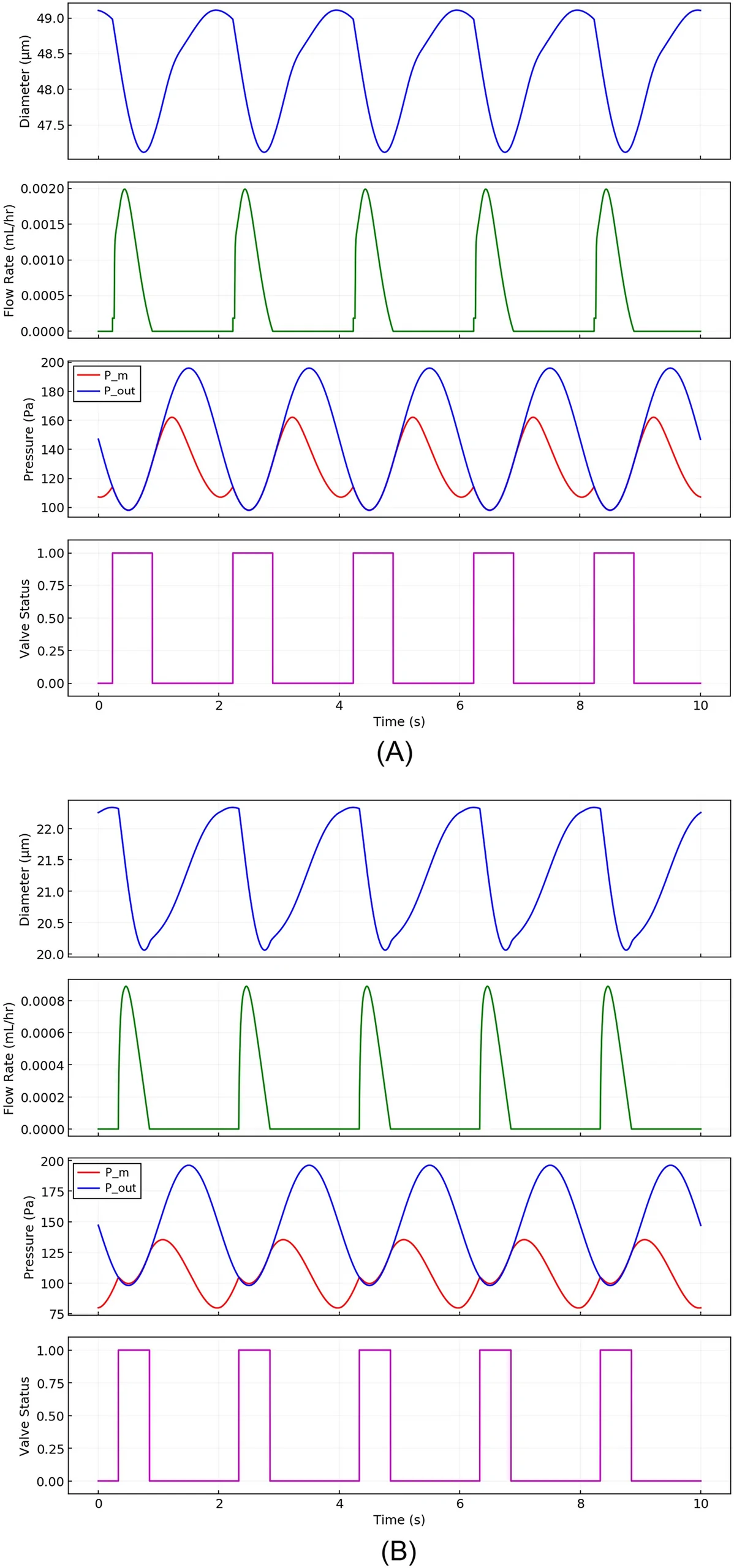
**A** 7-branched network, **B** 19-branched network. The figure shows outlet vessel diameters, lymph fluid flow rates, mid pressures, and valve status (open/closed) within branches 7 and 19 for concurrent (active and passive) force-driven lymphatic uptake

The effects of concurrent active and passive forces, along with the resultant vessel diameter, flow rate, pressure, and valve status, are shown in Fig. 10. It was assumed that there is a 90° phase difference between the active and passive pressure forces. The resulting outlet flow rates exhibit pulsatile characteristics, with cycle-mean and maximum flow rates of 3.77 × 10^-4^ ml/hr and 2.0 × 10^-3^ ml/hr for the 7-branch network, and 1.45 × 10^-4^ ml/hr and 8.8 × 10^-4^ ml/hr for the 19-branch network, respectively.

#### Parameter Sensitivity Analysis

**Fig. 11:**
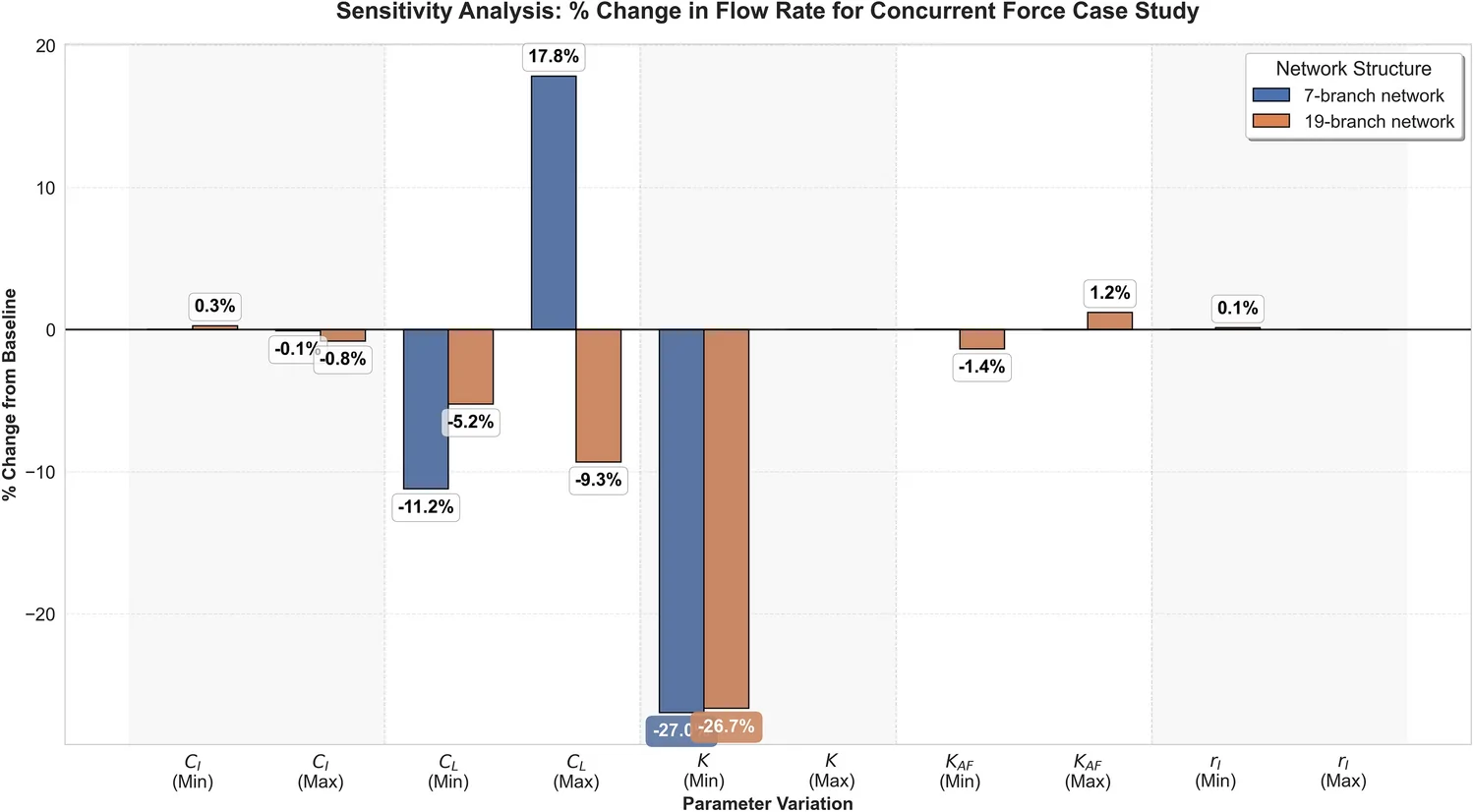
Parameter sensitivity analysis results for the active force-driven lymphatic uptake case study, showing the % change in flow rate relative to baseline

The concurrent force case yielded the highest baseline flow rate in the 19-branch network (1.45 × 10^-4^ ml/h). Similar to the active force-driven case study, the 7-branch network exhibited sensitivity to vessel wall compliance (C_L_), leading to a 17.83% increase in flow rate (Fig. 11). Permeability was the parameter that caused the most change in flow rate, with a maximum flow reduction of 27% (7-branch) and 26.7% (19-branch), comparable to the active pumping case. There was minimal influence of C_I_, K_AF_, and r_I_ in this concurrent force study.

## Global Sensitivity Analysis

In addition to the flow rate sensitivity analysis for baseline, minimum and maximum parameter values, we have conducted a global Sobol sensitivity analysis [38], providing a variance-based quantification of how each parameter contributed to uncertainty in lymphatic drainage across the different case studies. The findings were consistent with the one-at-a-time flowrate sensitivity trends but also revealed additional interaction effects (Table 3).

**Table 3:** First order (S₁) and total-order (S_T_) Sobol sensitivity indices for the key model parameters across the 7-branch and 19-branch lymphatic networks under the three pumping conditions

Parameter	Symbol	Network	Active	Passive	Concurrent
Vessel wall compliance	C_L_	7-branch	S_1_ = 1.00, S_T_ = 1.00	S_1_ = 0.23, S_T_ = 0.24	S_1_ = 0.92, S_T_ = 0.92
Vessel wall compliance	C_L_	19-branch	S_1_ = 0.81, S_T_ = 0.87	S_1_ = 0.05, S_T_ = 0.05	S_1_ = 0.70, S_T_ = 0.75
Interstitial space compliance	C_I_	7-branch	S_1_ = 0.00, S_T_ = 0.00	S_1_ = 0.00, S_T_ = 0.11	S_1_ = 0.01, S_T_ = 0.02
Interstitial space compliance	C_I_	19-branch	S_1_ = 0.00, S_T_ = 0.00	S_1_ = 0.00, S_T_ = 0.16	S_1_ = 0.04, S_T_ = 0.08
Interstitial space permeability	K	7-branch	S_1_ = 0.00, S_T_ = 0.00	S_1_ = 0.73, S_T_ = 0.82	S_1_ = 0.05, S_T_ = 0.07
Interstitial space permeability	K	19-branch	S_1_ = 0.00, S_T_ = 0.00	S_1_ = 0.63, S_T_ = 0.88	S_1_ = 0.18, S_T_ = 0.35
Radius (thickness) of the interstitium	r_I_	7-branch	S_1_ = 0.00, S_T_ = 0.00	S_1_ = 0.20, S_T_ = 0.20	S_1_ = 0.00, S_T_ = 0.00
Radius (thickness) of the interstitium	r_I_	19-branch	S_1_ = 0.00, S_T_ = 0.10	S_1_ = 0.00, S_T_ = 0.16	S_1_ = 0.01, S_T_ = 0.05
Spring constant of anchoring filaments	K_AF_	7-branch	S_1_ = 0.00, S_T_ = 0.00	S_1_ = 0.00, S_T_ = 0.00	S_1_ = 0.00, S_T_ = 0.00
Spring constant of anchoring filaments	K_AF_	19-branch	S_1_ = 0.13, S_T_ = 0.18	S_1_ = 0.00, S_T_ = 0.00	S_1_ = 0.06, S_T_ = 0.10

Under active pumping, variability in the model output is mostly governed by vessel wall compliance. In the 7-branch network, the first-order index approaches unity ${S}_{1,{C}_{L}}\approx 1.00$, indicating negligible interaction contributions from other parameters. The 19-branch network shows the same quantitative dominance of ${C}_{L}$ consistent with active pumping (Section "Active Force-Driven Lymphatic Uptake") being primarily regulated by vessel compliance.

The passive force-driven case showed that the output variance is dominated by interstitial permeability in both network configurations. Representative first-order indices are ${S}_{1,K}\approx 0.73$ for the 7-branches and ${S}_{1,K}\approx 0.63$ for the 19-branch network, confirming that tissue permeability is the principal determinant of drainage in the absence of active pumping.

The concurrent active case study results show that in the 7-branch network, ${C}_{L}$ It captures nearly all the variance, indicating that even with concurrent loading, vessel compliance remains the primary regulator of outflow. A similar pattern is observed in the 19-branch network, where ${C}_{L}$ continues to account for the majority of the variance, with only small additional contributions from ${K}_{AF}$, ${C}_{I}$, and ${r}_{I}$, confirming that vessel compliance remains the primary driver of uptake even in the more anatomically detailed model.

Interaction terms become more evident in the 19-branch network. Under passive forces, the total-order index for interstitial space permeability K exceeds its first-order index by roughly 0.25 (i.e. ${S}_{T,K}-{S}_{1,K}\approx 0.25$), indicating substantial multi-parameter interactions that augment the isolated effect of permeability. Moreover, interstitial compliance ${C}_{I}$ shows a near-zero first-order index but a non-zero total-order index, demonstrating that it contributes primarily through interactions rather than main effects. Together, these observations suggest a relationship between K and ${C}_{I}$: effective tissue hydraulic resistance is jointly regulated by permeability and pressure-dependent interstitial stiffness, and this coupling strengthens with network complexity.

## Discussion

Lymphatic drainage is crucial for maintaining interstitial fluid balance and overall tissue health. Both active and passive forces are expected to play vital roles in the absorption and transport of lymphatic fluid [39, 40]. The mechanisms driving interstitial lymphatic fluid uptake, however, are not fully understood, and scenarios like those investigated here are challenging to study *in vivo*. Given this challenging experimental context, the focus here was to use modelling to understand how active and passive forces impact lymphatic uptake and transport.

Due to the lack of direct experimental flow measurements in initial lymphatic networks, model validation was performed by comparing simulated flow rates with published computational studies. For the active case, our 7- and 19-branch models predicted cycle-mean outflows of 3.69 × 10^-4^ ml/hr and 1.37 × 10^-4^ ml/hr, respectively. These values fall within the range reported by Reddy and Patel [10] (10^-4^-10^-6^ ml/hr) for simpler capillary models and are lower than Li et al.’s (10^-3^ ml/hr) three-branch model [11]. While our branched networks introduce inherent differences in flow characteristics, the sensitivity analysis trends, in which vessel-related parameters dominate under the active force, align with physiological insights from Reddy and Patel. Additionally, our active case outflows are comparable to the lower end of collecting lymphatic flow rates reported by Dixon et al. [30] (8-19 × 10^-3^ ml/hr) and Blatter et al. [41] (3 × 10^-4^ ml/hr), supporting physiological plausibility.

For the passive case, results were compared with Ikhimwin [19], who modeled similar 7- and 19-branch networks. Their reported outflow rates ranged from 1 × 10^-6^ to 2.5 × 10^-4^ ml/hr, and our model estimated outflows well within this range. Sensitivity analysis indicated interstitium-related parameters, such as permeability, as dominant under the passive forces, which is in agreement with Ikhimwin [19]. These comparisons validate the model’s ability to capture physiologically relevant flow dynamics and highlight the influence of parameters in branched lymphatic networks.

Notably, both the maximum output flow rate and the total output flow are reduced in the 19-branch network compared to the 7-branch network. This can be attributed to smaller vessel diameters, which average 34.3 µm for the 7-branch network and 13.1 µm for the 19-branch network, as well as higher viscous losses due to having a higher number of small vessels and valves. Additionally, the average vessel lengths were 0.44 mm for the 7-branch network and 0.15 mm for the 19-branch network, resulting in 83% less surface area, contributing to the observed differences in flow characteristics. This observation suggests that optimal lymphatic architecture may vary by anatomical location, depending on local drainage requirements. The interstitial thickness (0.0003 m) and network topology data were estimated by Ikhimwin [19] based on imaging data. Although a constant interstitial thickness was used here, it is important to note that physiologically, interstitial thickness can vary among different lymphatic branches.

The active pumping mechanism and the passive force mechanism demonstrated comparable cycle-mean flow rates, particularly in the 7-branch network (3.69 × 10^-4^ ml/hr vs. 3.56 × 10^-4^ ml/hr). This demonstrates the physiological importance of passive forces, which are often considered secondary to active pumping. The passive mechanism, driven by external pressure changes, is effective, provided the interstitial environment is conducive to fluid movement. The slightly higher flow rate observed in the active case suggests that the intrinsic contractility provides an advantage in fluid transport.

The parameter sensitivity analysis revealed influence on total lymphatic drainage, with interstitial permeability (K) and vessel compliance (C_L_) emerging as the most important factors across all three mechanisms. One important finding is the higher sensitivity of the passive force mechanism to variations in interstitial permeability (K). A minimum value of K resulted in a near-complete cessation of flow, with reductions of 99.3% (7-branch) and 100.0% (19-branch). This highlights that the passive mechanism, which relies on the pressure gradient between the interstitial space and the vessel lumen, is heavily dependent on the permeability of the interstitial matrix. Pathological conditions that significantly reduces K, such as fibrosis or severe inflammation, could therefore effectively reduce passive lymphatic drainage.

While less pronounced, the active and concurrent case studies also showed sensitivity to K, with flow reductions of approximately − 26 to − 27% at the minimum K value. This demonstrates that, even when active pumping is present, the resistance of the interstitial space remains a governing factor in initial lymph uptake. Vessel compliance (C_L_) was the second-most influential parameter, particularly in the active and concurrent cases. Maximum C_L_ led to the largest flow increase in the concurrent case (+17.8% in the 7-branch network), while minimum C_L_ caused the largest flow reduction (− 11.2%). This is consistent with the physiological role of C_L_, where a more compliant vessel wall allows for greater volume change during active contraction and external pressure fluctuations, thereby increasing the net stroke volume and overall flow. The high sensitivity to C_L_ suggests that conditions leading to vessel stiffening (e.g. aging, chronic hypertension) could significantly impair lymphatic function, even if the active pumping frequency remains unchanged.

In contrast, the anchoring filament constant (K_AF_), interstitial compliance (C_I_), and interstitial thickness (r_I_) showed negligible influence on the cycle-mean flow rate across all mechanisms, with variations generally less than + /- 1.5%. The minimal impact of K_AF_ suggests that within the physiological range tested, the anchoring filaments are sufficiently robust to facilitate valve opening without becoming a rate-limiting factor for overall flow. Similarly, the flow appears relatively insensitive to variations in the volume of the interstitial space (C_I_) and the distance over which the pressure gradient acts (r_I_), suggesting that the primary resistance to flow is dominated by the vessel wall and the interstitial matrix permeability.

Across all three mechanisms, the 19-branch network resulted in a greater reduction in the absolute cycle-mean flow rate than the simpler 7-branch network (e.g. a reduction of 61-68% in all cases compared to the 7-branch). This is primarily due to the increased structural resistance and more valves and junctions in the 19-branch network, which collectively dissipate energy and reduce the efficiency of fluid transport, highlighting the impact of network topology in overall lymphatic transport capacity.

Interestingly, the relative sensitivity to the dominant parameters (K and C_L_) remained qualitatively similar between the two networks. This suggests that the fundamental mechanisms of lymph uptake are similar, regardless of the complexity of the network structure. However, the 19-branch network showed a slightly lower overall sensitivity to most parameters, implying that the increased structural resistance acts as a dampening factor, making the system more robust to small parameter variations.

The variance-based Sobol analysis provides additional insights regarding how the pumping mechanism and network complexity control lymphatic drainage. Under active pumping, drainage variability is governed almost entirely by vessel wall compliance ${C}_{L}$, with the 7-branch network showing ${S}_{1}$ near unity and the 19-branch exhibiting the same qualitative dominance, indicating that when contractile forcing is present, vessel distensibility is the principal regulator of output.

In contrast, the passive force case is dominated by interstitial permeability in both networks, and in the 19-branch configuration, the gap ${S}_{T}-{S}_{1}\approx 0.25$ signals meaningful interaction effects that amplify the isolated influence of permeability. The concurrent results available for the 7-branch network again point to C_L_ as the primary driver, suggesting that even modest active contributions can override interstitial control. Notably, ${C}_{I}$ carries a negligible first-order effect but a non-zero total-order index in the 19-branch passive case, supporting a relationship between K and C_I_: effective tissue hydraulic resistance reflects not only permeability but also pressure-dependent interstitial stiffness. Collectively, these findings support local trends which explain global interactions and highlight parameters most informative for future calibration and experimental targeting.

Additional experimental studies should be carried out to identify parameter-specific scaling based on physiological constraints. Here, we have used rodent data from different parts of the body, such as the skin and mesentery. Despite these limitations, the relative importance of the parameters identified remains valid, though effect magnitudes may be overestimated. It is important to note that different baseline conditions and parameter value ranges can show different outcomes.

The lymphatic bond graph model has several limitations. Simplified models were employed for secondary valves, and constant compliance values were assumed for the interstitium and vessel walls. In addition, it is worth noting that compliance values can be nonlinear due to strain-hardening or strain-softening [19], and interstitial permeability is not always constant, as the interstitium exhibits poroelastic properties [11]. Continuous pressure boundary conditions were used, which may not accurately represent discontinuous passive forces such as those generated by skeletal muscle contractions.

Future model extensions could include coupling it with a collecting lymphatic model, which could either be a 1D network model, another bond graph model, or a 0D model. Another extension would be to model disease states, considering changes in multiple parameters resulting from specific forms of lymphatic dysfunction. Furthermore, the model could incorporate blood capillary filtration to enhance its applicability and complexity. The developed model provides a framework for future organ-based lymphatic studies and explores lymphatic vessels across various parts of the body by adapting to physiological data. Cellular-level biomechanical models could also be integrated with collecting lymphatic vessels and combined with upstream image-derived structural information to provide a more complete multiscale representation of lymphatic function.

The simulation results confirm that all three mechanisms, active pumping, passive forces, and concurrent action, are capable of generating pulsatile flow and achieving net lymphatic uptake. The concurrent force mechanism consistently yielded slightly higher cycle-mean and peak flow rates in both the 7-branch (3.77 × 10^-4^ ml/hr) and 19-branch (1.45 × 10^-4^ ml/hr) networks. The assumed 90-degree phase difference between the active and passive pressure forces appears to slightly optimize (1-5%) the timing of vessel expansion and contraction relative to the external pressure, thereby maximising the net fluid movement.

This study successfully quantified the flow dynamics and parameter sensitivities of initial lymphatic uptake under active, passive, and concurrent force mechanisms. The results emphasize the roles of interstitial permeability (K) and vessel compliance (C_L_) in regulating lymph flow. Future work should focus on integrating these findings with in vivo data, particularly investigating the non-linear relationship between K and flow under pathological conditions, and exploring the optimal phase relationship between active and passive forces to further understand the maximum transport capacity of the initial lymphatics. Limitations of the current model include the assumption of a fixed phase difference and the simplified representation of the interstitial matrix, which could be addressed in future iterations.

## Supplementary Information

Below is the link to the electronic supplementary material.Supplementary file1 (DOCX 57 KB)

## Data Availability

Data are available from the corresponding author upon reasonable request.
