# Supplementary material for: A Modular Computational Approach for Assessing Active and Passive Force Contributions in Interstitial Lymphatic Fluid Uptake
Source: Ann Biomed Eng. 2026 Mar 7;54(9):3213–30. doi: 10.1007/s10439-026-04062-4 (PMC13522030; doi:10.1007/s10439-026-04062-4)
Supplement: Supplementary file 1 [file 10439_2026_4062_MOESM1_ESM.docx]

# Supplementary Materials

## A.1 Bond Graph Method

The bond graph method possesses three properties that enable the creation of a fundamental representation of any system in the human body and integration into a whole-body composite model. The first property is that bond graphs are acausal, which means they do not prescribe a fixed flow of causation from inputs to outputs. Instead, elements and their interconnections are represented by bonds and junctions, allowing for a flexible and interchangeable description of both energy storage and energy dissipation. Secondly, bond graphs are object-oriented, which allows equations to be systematically derived from their components and connections. Lastly, bond graphs are physics‑based and comply with conservation laws.

### A.1.1 Bond Graph Units and Junctions

In the bond graph approach, driving potentials (u), such as pressure and voltage, are expressed in Joules per unit of a specific quantity, while flows (v) represent that quantity per second. In engineering literature, the concept of ‘potential’ is referred to as ‘effort’. The Supplement follows standard bond‑graph conventions, where *u* denotes effort (pressure), *v* denotes flow, and *q* denotes the generalized displacement/charge variable. This notation is widely used in bond‑graph modelling and is retained here to allow readers to trace the energy‑based formulation. In the main manuscript, we use the more familiar physiological notation (P, Q, R) for readability. Both notations refer to the same underlying model.

Figure A1 illustrates an example effort or flow directed from element 1 to element 2. However, it does not represent causality but rather the sign convention for a particular variable. This quantity can vary across different physical systems, including meters, moles, coulombs, and so on. The small line in front of the arrow identifies it as a causal stroke (also called a power stroke or power bond). This represents the flow of power or energy in a particular direction within a system. Importantly, the units are defined so that the multiplication of effort and flow (u.v) yields power in Joules per second. This consistent definition across diverse physical systems establishes a unified modeling framework.

u(Jm^-3^)

v(m^3^s^-1^)

Element 1

Element 2

Figure A1: Schematic representation of a bond showing the energy flow.

To express a fluid mechanics problem using the bond graph formulation, four fundamental variables are required:

1. Potential (u), which represents pressure (J.m^−3^).

2. Flow (v), which denotes volumetric flow (m³.s^-1^).

3. The time integral of the potential (p), which represents momentum (J.s.m^-^³).

4. The time integral of the flow (q), which indicates quantity or volume (m³).

Furthermore, the bond graph method employs graphical representations to model physical systems. Two primary node types are utilized:

1. 0-junction: This node combines multiple flow bonds, ensuring a common potential (u) and enforcing mass conservation based on v.

2. 1-junction: At this node, multiple effort bonds merge, sharing a common flow (v) and enforcing energy conservation at the junction.

These concepts, the 0-junction and 1-junction, are essential for establishing conservation laws in bond graph modeling. The 0-junction ensures identical potential (u) at each port and enforces mass conservation based on v. Meanwhile, the 1-junction guarantees that flow (v) remains consistent at each port and enforces energy conservation based on potential (u). The sum of flows within a 0-junction is always zero, and the potential (u) remains constant.

### A.1.2 Bond Graph Elements

A distinctive feature of bond graph formulation is that it is modular and graphically interactive, which is achieved through bond graph elements. These elements serve as building blocks for a system representation. They also encapsulate empirical relationships with flow and potential, which may differ from the characteristics of physical systems.

To model lymph uptake into initial lymphatic vessels from the interstitial space, a fluid mechanical problem must be formulated, with pressure and flow rates as the main parameters. Two specific types of elements were used to construct the 7-branch model, as follows:

1. Capacitor (C) Element**:** C-elements or capacitor elements are used to model the energy stores in bond graph modeling. The capacitor element represents the storage of energy in the form of electrical charge or fluid pressure. However, of note, only static energy storage is considered:

|  | $u=\frac{q}{C}$ | (A.1) |
| --- | --- | --- |

where C is identified as compliance when considering a fluid mechanical system.

|  | $\dot{q}=v$ | (A.2) |
| --- | --- | --- |

2. Resistor (R) Element**:** R-elements or resistor elements represent the dissipation of energy or loss in a system, such as electrical resistance or frictional losses. Resistance elements are used to model viscous resistance in fluid dynamic systems.
   a. The flow resistance in the initial and pre-collecting lymphatic lumens is represented by Equation 1.
   b. Primary valve resistance is estimated using Equation 8.
   c. Flow resistance occurs due to porous properties of the interstitial space, as follows:

|  | $R_{I}=\frac{ln((2r_{i}+D)/D)\mu}{2\pi LK}$ | (A.3) |
| --- | --- | --- |

d. Secondary valve resistance is calculated based on Equation 9

Each of these elements and their interconnections form a bond graph, a graphical representation of the system dynamics.

Figure 3 also includes causal stroke perpendicular line segments in front or behind each bond (arrows), indicating the direction of the energy or power flow. Capacitance elements have a defined causality stroke position, which is located behind the arrow. The causality of the resistance bond depends on the other bonds meeting at 0- or 1-junction. These causal strokes are crucial when defining constitutive equations at the junctions.

#### *A.1.2.1 Equation of Anchoring Filament Force*

Force exerted by anchoring filaments, F_A_, can be calculated as [10]:

|  | $F_{A}=NK_{AF}(L_{f}-L_{f0})$ | (A.4) |
| --- | --- | --- |

where N is the number of filaments per unit length, K_AF_ is a constant related to the elastic properties of anchoring filaments (similar to a spring constant), L_f_ is the inter-fiber distance, and L_f0_ is the initial inter-fiber distance length which is 2.5 times the initial diameter (D_0_) [10]. The filament length L_f_ depends on the inter-fiber distance and the vessel wall’s instantaneous diameter (D), which is influenced by the interstitial fluid volume. Based on the Reddy and Patel [10] model, F_A_ can be calculated using:

|  | $F_{A}=NK_{AF}(\delta-\delta_{0})$ | (A.5) |
| --- | --- | --- |

where δ is the anchoring filament length and $\delta_{0}$ is the initial anchoring filament length. Then, Equation A.5 can be simplified considering the definition of $\delta$ and $\delta_{0}$from Reddy and Patel [10]:

|  | $\delta=\frac{L_{f}-D}{2}$ | (A.6) |
| --- | --- | --- |
|  | $\delta_{0}=\frac{L_{f0}-D_{0}}{2}$ | (A.7) |
| Considering the volume of the interstitial space and assuming the inter fiber length | | |
|  | $L_{f}=L_{f0}\left( \frac{v}{v_{0}} \right)^{0.5}=\left( \frac{r-D}{r-D_{0}} \right)$ | (A.8) |

Substituting A.7 and A.8 into Equation A.5 gives F_A_:

|  | $F_{A}=NK_{AF}(\frac{D_{0}-D}{2})(0.5\times\frac{L_{f0}}{\left( r-D_{0} \right)}+1)$ | (A.9) |
| --- | --- | --- |

## A.2 Bond Graph Modules

As mentioned in Section 2, we have created different modules to represent the different branches in the network. Here, we elaborate on the mathematical equations and the establishment of the connectivity between different modules.

### A.2.1 Lymphatic Blind Vessel Module

The Lymph Vessel Blind module represents a blind-ended initial lymphatic vessel (Q_in_=0), incorporating various physiological parameters and implementing the constitutive relations and conservation laws governing the behavior of the lymphatic vessel.

Ordinary Differential Equations (ODEs) and Key Relationships:

Wall flow rate change:

|  | $\frac{dv_{w}}{dt}=\frac{Q_{delay}-v_{w}}{\Delta T}$ | (A.10) |
| --- | --- | --- |

|  | $Q_{delay}=\frac{u_{w}-u_{m}}{R_{pv}}$ | (A.11) |
| --- | --- | --- |

Primary valve resistance:

|  | $R_{pv}=\frac{{HR}_{pv0}}{2\pi LD/d_{ec}\left( cosh \left( a\Delta u_{tm} \right)-1 \right)e^{-b\Delta u_{tm}}}$ | (A.12) |
| --- | --- | --- |

Transmural pressure difference:

|  | $\Delta u_{tm}=u_{w}-u_{m}$ | (A.13) |
| --- | --- | --- |

Vessel resistance:

|  | $R_{p}=\frac{64\mu L}{\pi D^{4}}$ | (A.14) |
| --- | --- | --- |

Vessel diameter:

|  | $D=2\sqrt{\frac{q_{m}}{\pi L}}$ | (A.15) |
| --- | --- | --- |

Change in fluid volume:

|  | $\frac{dq_{m}}{dt}=v_{w}-v_{p}$ | (A.16) |
| --- | --- | --- |

Mid-vessel pressure equation considering force balance:

|  | $u_{m}=\frac{0.5(q_{m}-q_{0})}{C_{L}}+u_{w}-u_{af}$ | (A.17) |
| --- | --- | --- |

The pressure force exerted by the anchoring Filaments:

|  | $u_{af}=\frac{NK_{AF}}{\pi D}\left( \frac{D_{0}}{2}-\frac{D}{2} \right)\left( \frac{{1.5D}_{0}}{r-D_{0}}+\frac{r}{r-D_{0}} \right)$ | (A.18) |
| --- | --- | --- |

Flow equation:

|  | $v_{p}=\frac{u_{m}-u_{out}}{R_{p}+R_{sv}}$ | (A.19) |
| --- | --- | --- |

Secondary Valve Resistance:

|  | $R_{sv}=\left\{ \begin{aligned} 0.025 dyn/s^{-1}{cm}^{5} if P_{m} \geq P_{out} \\ \infty if P_{m} < P_{out} \end{aligned} \right.$ | (A.20) |
| --- | --- | --- |

### A.2.2 Lymphatic mid-vessel module

The Lymph Vessel Mid module represents an intermediate segment of the lymphatic vessel, incorporating both inflow and outflow, as well as wall dynamics and valve functions.

Wall flow rate change (with a delay):

|  | $\frac{dv_{w}}{dt}=\frac{Q_{delay}-v_{w}}{\Delta T}$ | (A.21) |
| --- | --- | --- |

|  | $Q_{delay}=\frac{u_{w}-u_{m}}{R_{pv}}$ | (A.22) |
| --- | --- | --- |

Primary valve resistance:

|  | $R_{pv}=\frac{HR_{pv0}}{2\pi LD/d_{ec}\left( cosh \left( a\Delta u_{tm} \right)-1 \right)e^{-b\Delta u_{tm}}}$ | (A.23) |
| --- | --- | --- |

Transmural pressure difference:

|  | $\Delta u_{tm}=u_{w}-u_{m}$ | (A.24) |
| --- | --- | --- |

Vessel resistance:

|  | $R_{p}=\frac{64\mu L}{\pi D^{4}}$ | (A.25) |
| --- | --- | --- |

Vessel diameter:

|  | $D=2\sqrt{\frac{q_{m}}{\pi L}}$ | (A.26) |
| --- | --- | --- |

Flow equations (considering two secondary valves in the module):

|  | $v_{p1}=\frac{u_{in}-u_{m}}{0.5R_{p}+R_{sv1}}$ | (A.27) |
| --- | --- | --- |
|  | $v_{p2}=\frac{u_{m}-u_{out}}{0.5R_{p}+R_{sv2}}$ | (A.28) |

Flow rate at the inlet:

|  | $\frac{du_{in}}{dt}=2\frac{v_{L1}+v_{L2}-v_{p1}}{C_{L}}$ | (A.29) |
| --- | --- | --- |

Here, u_in_ represents the pressure at the junction connecting two lymphatic vessels, which have flow rates of v_L1_ and V_L2_.

Change in fluid volume:

|  | $\frac{dq_{m}}{dt}=v_{w}-v_{p2}+v_{p1}$ | (A.30) |
| --- | --- | --- |

Mid-vessel pressure equation considering force balance:

|  | $u_{m}=\frac{q_{m}-q_{0}}{C_{L}}+u_{w}-u_{af}$ | (A.31) |
| --- | --- | --- |

The force exerted by the anchoring Filaments:

|  | | $u_{af}=\frac{NK_{AF}}{\pi D}\left( \frac{D_{0}}{2}-\frac{D}{2} \right)\left( \frac{{1.5D}_{0}}{r-D_{0}}+\frac{r}{r-D_{0}} \right)$ | (A.32) |
| --- | --- | --- | --- |
|  | $R_{sv1}=\left\{ \begin{aligned} 0.025 dyn/s^{-1}{cm}^{5} if P_{m} \geq P_{out} \\ \infty if P_{m} < P_{out} \end{aligned} \right.$ | | (A.33) |
|  | $R_{sv2}=\left\{ \begin{aligned} 0.025 dyn/s^{-1}{cm}^{5} if P_{m} \geq P_{out} \\ \infty if P_{m} < P_{out} \end{aligned} \right.$ | | (A.34) |

### A.2.3 Lymphatic vessel-out module

There is no difference in the equations between the lymphatic mid and out-vessel modules. U_out_ is a boundary condition in the lymph vessel-out module, which is defined as a variable in the mid-vessel module.

Based on the case studies, U_out_ can be Equation A.35 or a constant value.

|  | $u_{out}=u_{ec}\cdot98.066\cdot\frac{1}{2}\left( 3-\cos\left( 2\pi f_{A}\left( t-t_{1} \right) \right) \right)$ | (A.35) |
| --- | --- | --- |

### A.2.4 Interstitial module

Interstitial resistance:

|  | $R_{I}=\frac{\mu\ln\left( \frac{2r+D}{D} \right)}{2\pi LK}$ | (A.36) |
| --- | --- | --- |

External pressure: Based on the case studies, U_e_ can be Equation A.37 or constant values.

|  | $u_{e}=u_{ec}\cdot98.066\cdot\frac{1}{2}\left( 3-\cos\left( 2\pi f_{P}t \right) \right)$ | (A.37) |
| --- | --- | --- |

Interstitial pressure ODE:

|  | $\frac{du_{w}}{dt}=\frac{v_{I}-v_{w}}{C_{I}}$ | (A.38) |
| --- | --- | --- |

Interstitial flow rate:

|  | $v_{I}=\frac{u_{e}-u_{w}}{R_{I}}$ | (A.39) |
| --- | --- | --- |

**Variable definitions:**

Based on the information provided, here is a comprehensive list of variables with their units for the lymphatic vessel and interstitial space models:

1. Time (t): second (s)

2. Fluid viscosity (μ): J⋅s/m³

3. Vessel length (L): meter (m)

4. Vessel diameter (D): meter (m)

5. Initial fluid volume (q_0_): m³

6. Vessel compliance (C_L_): m⁶/J

7. Primary valve resistance (R_pv_): J⋅s/m⁶

8. Poiseuille resistance (R_p_): J⋅s/m⁶

9. Secondary valve resistance (R_sv): J⋅s/m⁶

10. Flow rates (v_w_, v_p_, v_p1_, v_p2_, Q_delay_): m³/s

11. Pressures (u_w_, u_m_, u_out_, u_in_, u_e_): J/m³

12. Fluid volume (q_m_): m³

13. Anchoring filament stiffness (K_AF_): J/m²

14. Initial vessel diameter (D_init_): meter (m)

15. Number of anchoring filaments per unit length (N): m^-1^

16. Empirical constants (a, b): m³/J

17. Endothelial cell diameter (d_ec_): meter (m)

18. External pressure (u_ec_): J/m³

19. Frequencies (f_A_, f_P_): Hz

20. Radial distance (r): meter (m)

21. Permeability (K): m²

22. Interstitial resistance (R_I_): J⋅s/m⁶

23. Interstitial compliance (C_I_): m⁶/J

## A.4 Automated Model Generation and Simulation

This study employed Circulatory Autogen, an open-source software tool designed to streamline the process of combining CellML modules into comprehensive system models. This versatile tool facilitates the generation of coupled physiological models, such as neuron-to-cardiomyocyte interactions or patient-specific 0D blood flow networks and enables parameter identification for calibration against clinical data.

The CellML language, an open standard based on XML markup, was used to implement the mathematical equations of the modules. CellML also allows for the storage and exchange of computer-based mathematical models, enabling scientists to share models across different modeling tools. This standardization facilitates model reuse and accelerates model development by allowing components from one model to be easily incorporated into another.

To create a desired network using Circulatory Autogen, the key step is to define the vessel array in a CSV file. This file specifies the structure of your network by defining individual vessels and their connections. Each row in the CSV file represents a vessel, and includes information such as:

1. Vessel name and type

2. Input and output connections to other vessels

3. Specific parameters for each vessel type

The connections between vessels are established using input and output ports. Each vessel has defined input and output ports, which are used to connect it to other vessels in the network. For example, a vessel might have its output port connected to the input port of another vessel, creating a flow path through the network. To create complex networks, you can define branching points where one vessel's output connects to multiple input ports of other vessels. Similarly, you can create convergence points where multiple vessels' outputs connect to a single vessel's input.

The Circulatory Autogen tool [27] uses this vessel array definition to automatically generate the necessary CellML files, ensuring that all connections are properly established and that the mathematical equations for each vessel are correctly implemented. This approach allows for the creation of intricate networks while maintaining the modularity and reusability of individual vessel components.

By modifying the vessel array CSV file, you can easily adjust the network structure, add or remove vessels, and change connections between vessels. This flexibility enables the creation of a wide range of physiological models, from simple linear systems to complex, branching networks representative of various circulatory systems.

For detailed instructions on creating the vessel array file, defining vessel types, and establishing connections, refer to the comprehensive Circulatory Autogen documentation available https://github.com/FinbarArgus/circulatory_autogen. This resource provides in-depth guidance on leveraging the full capabilities of the software for complex physiological modeling tasks.

### A.4.1 Sobol Variance-Based Sensitivity Analysis

This section provides the mathematical formulation and computational procedure used to compute the Sobol sensitivity indices reported in the main text. The model output used for sensitivity analysis is the cycle-averaged lymphatic outflow rate at the outlet branch (branch 7 for the simple network; branch 19 for the complex network).

**Variance Decomposition Framework:** Let the model output be defined as Y = f(θ), where θ = (θ₁, θ₂, …, θ_k) and each parameter θᵢ is sampled independently over its physiological range. The Sobol method decomposes the total output variance into additive contributions from individual parameters and their interactions:

|  | $Var\left( Y \right)=\Sigma_{i=1}^{k}V_{i}+\Sigma_{i<j}V_{ij}+\Sigma_{i<j\mathcal{<l}}V_{ij\mathcal{l}}+\ldots+V_{1,2,\ldots,k}$ | (A.40) |
| --- | --- | --- |

**Sobol Sensitivity Indices:** First-order (main-effect) and total-order indices are defined as follows:

|  | $S_{i}=V_{i}/Var\left( Y \right)=Var_{\theta_{i}}\left( E\left( Y \vert\theta_{i} \right) \right)/Var\left( Y \right)$ | (A.41) |
| --- | --- | --- |
|  | $S_{T_{i}}=1-Var_{\theta_{\sim i}}\left[ E\left( Y \vert\theta_{\sim i} \right) \right]/Var\left( Y \right)$ | (A.42) |

**Sampling and Numerical Estimation (Saltelli Scheme):** We employed Sobol quasi-random sequences with the Saltelli extension to construct two base sampling matrices A and B, and k hybrid matrices A_B^{(i)} formed by swapping the i-th column of A with the i-th column of B. The model is evaluated at all rows of A, B, and each A_B^{(i)}. The total number of model evaluations per pumping condition is:

|  | $N_{e}val=N\left( k+2 \right)$ | (A.43) |
| --- | --- | --- |

**Estimator Formulae:** The following numerically stable estimators were used for first- and total-order indices:

|  | $S_{i}=\left[ \left( 1/N \right)\Sigma_{n=1}^{N}f\left( B^{\left( n \right)} \right)\left( f\left( A_{B}^{\left( i \right)\left( n \right)} \right)-f\left( A^{\left( n \right)} \right) \right) \right]/Var\left( Y \right)$ | (A.44) |
| --- | --- | --- |
|  | $S_{T_{i}}=\left[ \left( 1/N \right)\Sigma_{n=1}^{N}\left( f\left( A^{\left( n \right)} \right)-f\left( A_{B}^{\left( i \right)\left( n \right)} \right) \right)^{2} \right]/\left( 2Var\left( Y \right) \right)$ | (A.45) |

Implementation follows the circulatory_autogen sensitivity-analysis module [27], with parameters sampled independently over the physiological ranges in Table 2 of the main text. Sensitivity was computed separately for passive, active, and combined pumping conditions. Output Y is the cycle-averaged lymphatic outflow rate at the outlet branch. Convergence was checked by increasing N until rank ordering of S_i_ and S_Ti_ stabilized.
